# Into the Himalayan Exile: The Phylogeography of the Ground Beetle *Ethira* clade Supports the Tibetan Origin of Forest-Dwelling Himalayan Species Groups

**DOI:** 10.1371/journal.pone.0045482

**Published:** 2012-09-26

**Authors:** Joachim Schmidt, Lars Opgenoorth, Steffen Höll, Ralf Bastrop

**Affiliations:** 1 Faculty of Geography, University of Marburg, Marburg, Germany; 2 Department of Ecology, University of Marburg, Marburg, Germany; 3 Institute of Biological Sciences, University of Rostock, Rostock, Germany; American Museum of Natural History, United States of America

## Abstract

The Himalayan mountain arc is one of the hotspots of biodiversity on earth, and species diversity is expected to be especially high among insects in this region. Little is known about the origin of the Himalayan insect fauna. With respect to the fauna of high altitude cloud forests, it has generally been accepted that Himalayan lineages are derived from ancestors that immigrated from Western Asia and from adjacent mountainous regions of East and Southeast Asia (immigration hypothesis). In this study, we sought to test a Tibetan Origin as an alternative hypothesis for groups with a poor dispersal ability through a phylogeographic analysis of the *Ethira* clade of the genus *Pterostichus*. We sequenced COI mtDNA and the 18S and 28S rDNA genes in 168 Pterostichini specimens, including 46 species and subspecies of the *Ethira* clade. In our analysis, we were able to show that the *Ethira* clade is monophyletic and, thus, represents a Himalayan endemic clade, supporting endemism of two of the basal lineages to the Central Himalaya and documenting large distributional gaps within the phylogeographic structure of the *Ethira* clade. Furthermore, the molecular data strongly indicate very limited dispersal abilities of species and subspecies of these primary wingless ground beetles. These results are consistent with the hypothesis of a Tibetan Origin, which explains the evolution, diversity and distribution of the Himalayan ground beetle *Ethira* clade much more parsimoniously than the original immigration hypothesis.

## Introduction

The Himalayan mountain arc is one of the hotspots of biodiversity on earth, which has been impressively demonstrated in plants and vertebrates [1]. The species diversity on tropical mountains is expected to be especially high for insects [2], [3]. However, due to the lack of comprehensive revisions of the most diverse taxa, such as the Coleoptera, Diptera and Hymenoptera, it is impossible to estimate the number of insect species occurring in the Himalayan mountain arc. Furthermore, little is known about the reasons underlying the remarkably high diversity, mechanisms of speciation, or origin of the Himalayan insect fauna. Until recently, most contributions addressing these topics were focused on species groups with a high dispersal ability, such as butterflies and flying beetles [4]–[8]. It has generally been accepted that Himalayan lineages of the cloud forest fauna are derived from ancestors that immigrated from Western Asia and from adjacent mountainous regions of East and Southeast Asia, respectively (see [9] for an overview). These results generally agree with findings in birds [10], [11]. However, based on a more comprehensive avifaunal study, Weigold [12] proposed Tibet as an independent centre of evolution in the course of its uplift as early as 1930, although this hypothesis had largely been ignored due to the late post-mortem publication of his work in 2005. An intensive exploration of the ground beetle family Carabidae carried out in High Asia during the past two decades provided evidence of an origin of the Himalayan cloud forest fauna in South Tibet (e.g. [13], [14], [15]). As a result of these morphology-based studies, at least four observations were made that are difficult to explain solely based on immigration of ancestral species from adjacent eastern and western regions followed by dispersal of the descendants along the High Himalayan mountain chain and subsequent diversification:

1. Various Himalayan endemic ground beetle species groups were identified showing no close relationships to lineages occurring in areas adjacent to the Himalaya (see [15] for an overview). Following the immigration hypothesis, for each of these endemic species groups, the complete extinction of all lineages within the vast mountainous areas that seamlessly border the northwest, north (Hindukush, Pamir, Tian Shan) and east of the Himalaya (the eastern slopes of the Tibetan Plateau in western China and Indochina) would have to be assumed.
2. As a special case of (1) several putatively older lineages of Palearctic ground beetle taxa have been detected that are endemic to the high montane forest zone of the Central Himalaya, (e.g., the *Calathus heinertzi* group, *Lepcha*, *Nepalobroscus*, respectively [16]–[18]). Following the immigration hypothesis, in addition to the extinction scenario described in (1) the complete extinction of relatives in the eastern and in the western parts of the High Himalayan mountain arc must be considered to have occurred in all of these cases. However, there are no climatic or other abiotic reasons for these isolated ranges, as can easily be inferred from the fact that this pattern is not observed among good dispersers, such as plants, birds and butterflies.
3. Among the most striking zoogeographical phenomena of the Himalaya are the wide gaps within the distributional areas of several wingless ground beetle lineages, e.g. the *Carabus* subgenus *Meganebrius* [19] and the *Ethira* clade of the genus *Pterostichus* [14]. Both of these groups include representatives in the Northwest Himalaya and in the Central Himalaya, but no relatives in the Kumaon Himalaya. Climatically, this mountain area is a transitional area between the drier Northwest and the mistier Central and East Himalaya, and no ecological factors are known that could explain the absence of representatives of the taxa named above. Other carabid lineages have representatives throughout the Western Himalaya, including the Kumaon Himalaya and the Eastern Himalaya, but no relatives across wide regions of the Central Himalaya, e.g. *Chaetobroscus* [20], [21], *Pristosia* [15], and the *Calathus wittmerianus* group [19].
4. Morphology-based analyses in numerous Himalayan arthropod taxa indicate extremely limited distributional ranges of these species and the occurrence of strictly allopatric speciation (see [9] for an overview). The most striking cases are known from high altitude ground beetles: The distributional areas of primary wingless Carabidae species are commonly restricted to single slopes or valleys, whereas the most closely related species occur in directly adjacent areas of the mountains (e.g. [14]–[16], [22], [23]). These observations of local endemism among all of the primary wingless high altitude ground beetle groups strongly argue against the occurrence of long-distance dispersal during the evolutionary history of these lineages.

Based on these findings, Schmidt [14] hypothesised that during the early phase of mountain uplift, the southern part of the Himalayan-Tibetan Orogen was an independent centre of evolution that was well separated from other mountainous regions. Adaptation to high altitude environments and primary diversification within the groups occurring in these areas would have taken place in the Tibetan Himalaya (Inner Himalaya, Tethys Himalaya) and in the Transhimalaya, respectively, long before the final uplift of the High Himalayan mountain chain. Colonisation of the High Himalayan mountain chain would have occurred in the course of its growth via ancestral species coming from the north. Due to orogenesis resulting in additional uplift and intensive drying of Tibet due to its location in the rain shadow of the High Himalaya (a process that is well documented in the geoscience literature, [24]–[26], the primary distributional areas of all forest dwelling lineages in South Tibet were lost. An extensive stepwise extinction process is hypothesised to have taken place in the Inner Himalaya, rather than multiple extinctions along the High Himalayan mountain arc. This alternative model of Himalayan faunal genesis seems to explain all of the distributional anomalies of the Himalayan ground beetle fauna, without any additional assumptions being necessary.

The aim of this study is to test this Tibetan origin hypothesis for a species-diverse Carabidae taxon as a model for groups with a poor dispersal ability through a phylogeographic analysis and to explore what the major speciation processes are within wingless ground beetle lineages. More specifically, using sequence data from COI mitochondrial DNA (mtDNA) and nuclear 28S and 18S ribosomal DNA (rDNA) from 46 species and subspecies of the *Ethira* clade of the genus *Pterostichus* and 95 species of various other Pterostichini taxa, we aim to test i) whether the *Ethira* clade is monophyletic and, thus, a Himalayan endemic clade; ii) whether Central Himalayan endemism is supported for basal lineages by the molecular analyses; iii) whether distributional gaps become apparent within the phylogeographic structure of the *Ethira* clade; and iv) whether the assumption of a strongly limited dispersal ability of the modern species and subspecies of this group can be verified by the molecular data. Finally, we will discuss whether the results of the phylogeographic analysis are consistent with the assumption of a Tibetan origin for the *Ethira* clade. In performing this work, we benefited greatly from the previous studies carried out by Sasakawa & Kubota [27], [28] and of Will & Gill [29], who studied several other Pterostichini ground beetle groups, thus providing a comprehensive set of DNA sequences for comparison.

## Materials and Methods

### The *Ethira* clade: Summary of Previous Morphological, Biogeographical and Ecological Data

**Figure 1:**
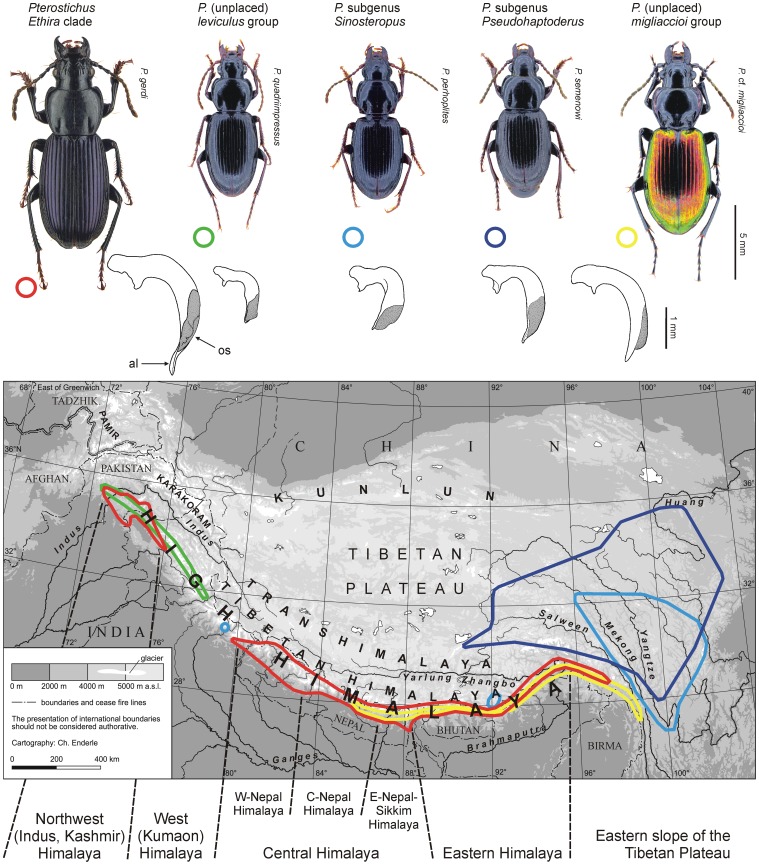
Map of High Asia. The showing main parts of the Himalayan Tibetan mountain system, main ecological divisions of the High Himalayan mountain arc, and distribution of five species groups of primary wingless *Pterostichus* ground beetles (areas framed by coloured lines): red, *Ethira* clade, a Himalayan endemic with an extensive distributional gap across the Kumaon Himalaya; green, *Pterostichus* (unplaced) *leviculus* group, a Northwest Himalayan endemic; light blue, subgenus *Sinosteropus*, with more than 30 species and subspecies on the eastern Tibetan Plateau, with a single species in the Western Himalaya, and with two species in a restricted area of the eastern Tibetan Himalaya; dark blue, subgenus *Pseudohaptoderus*, endemic to the eastern Tibetan Plateau; yellow, *Pterostichus* (unplaced) *migliaccioi* group, an East Himalayan endemic (distributional data after [14,30,60,61,62 and unpublished data]). General habitus and median lobe of the male genital of typical representatives of the relevant species groups are figured above. Abbreviations: al  =  apical lamella; os  =  ostium of aedeagal median lobe.

Based on morphological data, the first author [14] assumed monophyly of a group encompassing both the taxa *Ethira* Andrewes, 1936, which has ten species in the Northwest Himalaya, and *Pseudethira* Sciaky, 1996, which has a much larger species number in the Central and Eastern Himalaya. In his latest review, Schmidt [30] noted a total of 78 *Pseudethira* species and subspecies. The relatively long median lobe of the male genitals of *Ethira* and *Pseudethira* with markedly developed apical lamella (Figure 1) was hypothesised to be a synapomorphic feature of these taxa and, thus, defines the *Ethira* clade. All other Pterostichini species groups occurring the Himalaya and adjacent mountains exhibit quite different male genital shapes. Nevertheless, in contrast to the opinion of previous authors who treat *Ethira* as a separate genus due to several striking anomalies in its habitus characters (last by [31]), this male genital morphology also leads to the assumption of a systematic position of the *Ethira* clade well within the “modern *Pterostichus*” ( =  Pterostichini *sensu* Jeannel 1942), a species group showing a deflected ostium of the median lobe. Although Bousquet [32] believed that the phylogenetic importance of this character was overestimated, the monophyly of this group was supported by a molecular analysis performed by Sasakawa & Kubota [27] using two nuclear gene sequences (wingless and 28S rDNA). The majority of the species hitherto included under the genus *Pterostichus (e.g.* [32]–[34]) appear to belong to the “modern *Pterostichus*”, which present a male genital ostium +/− bent markedly to the left (approx. 1,000 Holarctic species). Within this highly diverse group, the sister group of the *Ethira* clade remains unknown at present.

All species belonging to the *Ethira* clade live at high altitudes. The distributional area of the group ranges from the mountains of the northern Indus catchment along the High Himalayan mountain arc to the mountains of the eastern Brahmaputra catchment (Figure 1). A large distributional gap in the Kumaon Himalaya divides the distributional area of the subgenus *Ethira* from that of the subgenus *Pseudethira*. Within *Pseudethira*, Schmidt [14] defined eight subgroups based on morphological data, which are all endemic to restricted parts of the Himalaya. Several new species were later added to these subgroups (see overview in [30]). All species of the *Ethira* clade are primary wingless (winglessness by descent). This is indicated not only by absence of hind wings but also by irreversible markedly shortening of metathoracal plates among all species of that clade. Their larvae and imagines (the latter having a body length of 11–18 mm) are night-active predators of small invertebrates. They inhabit humid soils and leaf litter of mountain cloud forests or alpine meadows, and they hide under large stones, rotten trunks or in humid crevices during the day. These species are strictly adapted to temperate or even alpine environments and to relatively humid habitat conditions. Thus, species of the *Ethira* clade are lacking in the subtropical valleys of Himalayan foothills as well as in the dry valleys of the Inner Himalaya. The habitat preference characteristics and winglessness of these beetles both seem to strongly limit their distributional ability. It is therefore not surprising that the distributional areas of all of the known species and subspecies of the *Ethira* clade each are very limited and usually do not exceed a single mountain slope or valley system.

### Taxon Sampling, Identification and Classification

The sampling method used in this study was designed to test species and subspecies boundaries as well as species group monophyly. We focused on three taxon sampling strategies (Table S1). First, to determine the taxonomic position of the *Ethira* clade, we considered all subgenera of *Pterostichus* and putative closely related taxa for which DNA sequence data for the selected 28S rDNA and 18S rDNA gene segments with sufficient lengths were available in GenBank. We added to this taxon set members of most of the subgeneric *Pterostichus* groups and putative closely related taxa that are known to occur in the Himalaya and in immediately adjacent areas of Central and Eastern Tibet. In addition to *Ethira* and *Pseudethira*, these taxa are *Aristochroa*, *Lesticus, Nirmala, Trigonognatha, Trigonotoma*, the *Pterostichus* subgenera *Bothriopterus, Circinatus, Pseudohaptoderus,* and *Sinosteropus* and the *P. migliaccioi* species group, which presumably represents an undescribed supraspecific taxon. Altogether, we included 43 *Pterostichus* taxa at the subgeneric level and 13 putatively related genera in this analysis. One West Himalayan endemic group (the *P. leviculus* species group, Figure 1) and several *Pterostichus* subgenera endemic to the eastern Tibetan Plateau (e.g., *Gutta, Morphohaptoderus, Neohaptoderus, Straneostichus, Tschitscherinea*) could not be considered here due to the lack of sufficiently conserved material to allow DNA sequence data to be obtained. However, species of all of these taxa differ markedly in terms of habitus and male genital characters, and thus, closer relationships to the *Ethira* clade can be excluded.

Second, to ensure species group monophyly and to identify evolutionary lineages within the species group, we included as many taxa of the *Ethira* clade as possible. This sampling was also limited by the availability of sufficiently conserved material. We were able to analyse 46 species and subspecies belonging to the *Ethira* clade, including samples of all of the species groups defined by the previous morphological study carried out by Schmidt [14].

Third, to test species and subspecies boundaries and morphological taxonomy for selected species of the *Ethira* clade, we considered individuals from different localities within the entire species range together with individuals from all sympatric species and from all parapatric species. A total of 142 specimens from 75 sampling sites of *Ethira* clade species were therefore investigated.

The resources used to identify specimens are listed in Table S1. The classification of species into subgenera and genera in the tribe Pterostichini followed Lorenz [34]. The new sequences obtained have been deposited in GenBank under accession numbers JX535618–JX535765 and JX560814–JX560962.

### Sequence-data Acquisition Methods

Genomic DNA samples were prepared from specimens conserved in the field in 70–98% ethanol and later preserved in absolute EtOH and stored at −20°C. In a single case, DNA was prepared from a dried specimen. DNA was extracted from femoral or thoracic muscles using a silica spin column procedure with NucleoSpin® Tissue Kit (Macherey-Nagel, Düren, Germany) following the protocol provided by the manufacturer.

Approximately 1440 bp of the COI mitochondrial region, a sequence segment of 1020–1080 bp of the D1–D3 region of 28S rDNA, and a sequence segment of 1920–1980 bp of 18S rDNA were amplified via the polymerase chain reaction (PCR) in either a Flex Cycler (Analytic Jena, Jena, Germany) or a TGradient Cycler (Biometra, Göttingen, Germany) using Molzym Taq polymerase and the basic protocols recommended by the manufacturers. The primers and details of the cycling reactions used are presented in Tables S2 and S3.

PCR products were purified using the NucleoSpin® Extract Kit (Macherey-Nagel) or the innuPREP Gel Extraction Kit (Analytik Jena) and were then sequenced using the DTCS Quick Start Kit (Beckman Coulter, Krefeld, Germany) and subjected to electrophoresis in an automated DNA sequencer (CEQ™ 8000; Beckman Coulter). The sequences were automatically analysed using the software CEQ™ 8000 (Beckman Coulter) and manually edited and aligned using BioEdit software [35]. All variable positions within a sequence were checked on the basis of the corresponding electropherogram.

### Sequence Alignment

The COI sequences were aligned using MUSCLE [36] with default settings as implemented in MEGA 5.05 [37]. Alignment on the basis of nucleotides and amino acids produced similar results, as no ambiguities, such as insertions, deletions or stop codons, were found. Ribosomal RNA (rRNA) 18S and 28S sequences were aligned using their secondary structure with RNAsalsa 0.8.1, as outlined in Stocsits et al. [38]. As the initial input, we used constraint files based on the secondary structures of *Bembidion chalceum* 18S rRNA downloaded from www.rna.ccbb.utexas.edu (EF648647) and *Apis mellifera* 28S rRNA downloaded from http://zfmk.de/web/Forschung/Abteilungen/AG_Wgele/Software/RNAsalsa/index.en.html. These sequences were aligned with our dataset using the MUSCLE algorithm in MEGA 5.05 [37]. Starting with this initial alignment and the constraint file, RNAsalsa implements a workflow for both RNA secondary structure prediction and enhanced structural alignment that results in a final multiple sequence alignment together with a consensus structure. To exclude ambiguously aligned sites, we used Aliscore v1.0 (downloaded at www.zfmk.de/web/Forschung/Abteilungen/AG_Wgele/Software/Aliscore/index.en.html), treating gaps as ambiguities (option -N), using a windowsize of 6 (option -w 6) and comparing all sequences pairwise (option -r 100000). Other parameters were kept at default settings.

### Phylogenetic Analyses

Substitution models for the COI gene and for the loop regions of the 18S and 28S genes were chosen following the approach of Minin *et al.* [39], as implemented in the Perl scriptDT-ModSel. In contrast to the likelihood ratio tests implemented in MrModeltest 2.2 [40], this approach of performance-based model selection uses the Bayesian information criterion and considers the relative branch-length error as a performance measure in a consistent framework with a penalty for overfitting based on decision theory [39]. DT-ModSel selected the GTR I+Γ model for the COI mtDNA data, the 18S rRNA loop region and the 28S rRNA loop region. For the rRNA stem regions, the doublet model proposed by Schoniger & von Haeseler [41] was assigned in the Bayesian analysis. For this procedure, unambiguous stem pairs were derived on the basis of the consensus structure from RNAsalsa and specified in the MrBayes input file.

MrBayes was run for 10 million generations, sampling trees every 1000^th^ generation and using a random tree as a starting point. Inspection of the standard deviation of split frequencies after the final run indicated convergence of Markov chains (at least <0.05). In all analyses, two parallel Markov chain Monte Carlo simulations with four chains (one cold and three heated) were run. The first 10% of the samples of each run were discarded as burn-in. Based on the sampled trees, consensus trees were produced using the sumt command in MrBayes.

Bayesian analyses were conducted on five nucleotide matrices. In addition to three individual gene matrices, one matrix was formed by the combination of the 18S and 28S rDNA sequence data (“18S + 28S dataset”), and one matrix was formed by the combination all three genes (‘‘Ribo + COI dataset’’). An incongruence length difference test was performed in PAUP prior to the combination of the three nucleotide datasets. All five nucleotide sets were also analysed in a maximum likelihood framework using MEGA 5.05 [37]. It is not possible to implement the doublet model in MEGA – thus all positions of the rDNA alignments were treated under the same evolutionary model. In a final approach the Bayesian tree was used as constrain in a maximum likelihood analysis to derive ML bootstrap support for the phylogenies.

### Evolutionary Rates and Age Estimation

No suitable fossil records currently exist to properly calibrate the *Ethira* clade or even the genus *Pterostichus*. *Pterostichus* fossil records are known only from Baltic Amber. Klebs [42] listed five specimens of *Pterostichus* spec. and one specimen near *Pterostichus* in his collection of Baltic Amber inclusions. In addition, a photograph of a Baltic Amber fossil of a presumably “modern *Pterostichus*” species with a habitus similar to recent species of the subgenus *Lyrothorax* was presented by Weitschat & Wichard [43]. However, the true systematic position of these fossils will remain uncertain until a comprehensive morphological analysis is performed. Therefore, our calibration is provisional. We use a first rough calibration point in our analysis, setting the minimum age for the *Pterostichus* genus to 35–42 million years (Kaplan et al 1977, as cited by [44]).

As an additional preliminary calibration point, we used the Euro-Alpine orogenesis. The Euro-Alpine *Pterostichus* clade (comprised of *Pterostichus fasciatopunctatum* and *P. ebenus* in our COI dataset) must have evolved after high montane forests had been established as these species are bound to high montane habitats. High montane forests in Europe are first described at approximately 15 mya [45].

Finally mutation rates are available from other published beetle studies. These rates vary to a great degree ranging from 0.0038 mutations per million years reported for Chrysomelidae [46] and 0.0046 mutation per million years in the closely related ground beetle tribe Sphodrini [47], to 0.0152 mutations per million years in the genus *Trechus* on the Canary Islands [48]. In order to assess the impact of the different calibration points and mutation rates we implemented four different approaches to our dating: using i) both calibration points as well as the available mutation rates, ii) both calibration points, iii) the respective calibration points, and iv) the available mutation rates.

In all approaches we subjected the mtDNA data to a Bayesian Markov chain Monte Carlo (MCMC) analysis to estimate approximate divergence times for the *Ethira* clade using an uncorrelated lognormal relax molecular clock analysis method [49], as implemented in BEAST v.1.7.1 [50]. Input files were generated with BEAUTi 1.7.1 [51]. In all four approaches we used an uncorrelated lognormal relaxed molecular clock under the T93+I + Γ model of nucleotide substitution. A random tree was used as the starting tree considering the Yule process tree prior.

The mutation rates were implemented with a lognormal prior distribution, a mean of 0.0046 (derived from the closely related *Sphodrini*) and truncated to 0.0152 (upper bound) and 0.0038 (lower bound) mutations per million years (see above).

The *Pterostichus* calibration was implemented by applying a lognormal distribution with an offset of 35 mya as a prior with a standard deviation of 1. For the Euro-Alpine calibration we chose a normal distribution to allow for uncertainties in orogenetic calibration, using 15 mya as a prior with a standard deviation of 3 (95% range: 10–20 mya).

All runs were performed with 50 million generations, sampling every 5000th generation and burn-in set to 10% of the samples. The results were displayed in Tracer v.1.7.2 [51] to check for stationarity. Tree information was annotated with TreeAnnotator v.1.7.1 [50] and visualised in FigTree v.1.1.2 [50].

Both calibration points must be considered to be very rough approximations and should be complemented with more thorough investigations in the future regarding both Baltic Amber and the phylogeographic treatment of the extremely species-rich Euro-Alpine *Pterostichus* fauna.

## Results

### Monophyly and Systematic Position of the *Ethira* clade

**Figure 2:**
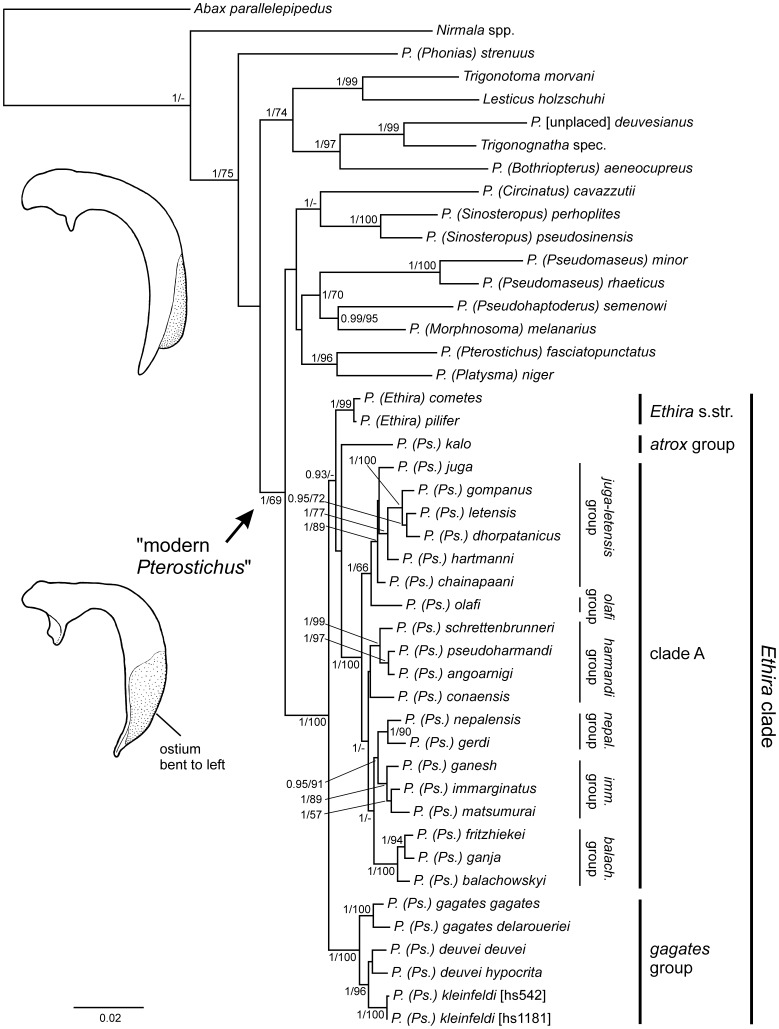
Results of the combined 28S + 18S rDNA + COI mtDNA data analysis. The 50% majority rule consensus tree of the Bayesian analysis. of the combined 28S + 18S rDNA + COI mtDNA sequence data set of Pterostichini (*Ethira* clade, 10 additional *Pterostichus* subgeneric taxa, and 5 putatively related genera) using *Abax parallelepipedus* as outgroup taxon. Numbers on or beside branches are Posterior Probabilities ≥90%/Bootstrap support values. Numbers in brackets refer to the internal specimen identification code and are added in cases when more than one specimen of a morphological species was added to the analysis. The deflected median lobe of male genital of the “modern *Pterostichus*” with ostium turned to the left is exemplary figured beside the relevant subtree; the presumably plesiomorphic shape with ostium in dorsal position is figured above. Beside the *Ethira* clade four of its basal lineages and six terminal species groups of clade A are designated and are discussed in the text. Abbreviations: *balach*.  =  *balachowskyi*; *imm*.  =  *immarginatus*; *nepal*.  =  *nepalensis*; *P.  =  Pterostichus; Ps.  =  Pseudethira*.

The monophyly of the *Ethira* clade is strongly supported by the combined Ribo + COI dataset (Posterior Probabilities/Bootstrap support 1/100, Figure 2), by the 18S + 28S dataset (1/99, data not shown), as well as by the 28S and COI data alone (Figures S1, S3). Separate analysis of the 18S rDNA data did not support the monophyly of the *Ethira* clade. However, this tree failed to support clades at even higher taxonomic levels, such as the tribe Pterostichini, when using an extended dataset with several Harpalinae + *Bembidion chalceum* as outgroup taxa (not shown).

The systematic position of the *Ethira* clade within the “modern *Pterostichus*” is also supported by the combined Ribo + COI and 18S + 28S datasets, with Posterior Probabilities of 1 and 0.98, respectively. This clade encompasses all of the species in our dataset with male genitals with a deflected ostium and the *Ethira* clade was observed to be one of the most recently evolved lineages (Figure 2). The clade of “modern *Pterostichus*” is also seen in the 28S tree, which is based on a much larger number of *Pterostichus* subgenera and putatively related taxa (Figure S1). This clade did not receive sufficient support in the separate 28S rDNA analysis. Nevertheless, the systematic position of the *Ethira* clade within the “modern *Pterostichus*” appears to be confirmed by this analysis as well, as it is part of a well-supported lineage (Posterior Probability of 0.99) comprised of the Euro-Alpine *Pterostichus* (*Oreophilus, Platypterus, Pterostichus* s.str.), some East Asian endemics (*Circinatus, Georgeballius, Sinosteropus*) and additional Holarctic *Pterostichus* subgenera. The taxonomic position of the *Ethira* clade was not resolved by analysis of the COI dataset alone. Other than some exceptions (*Aristochroa + Trigonognatha, Lesticus + Trigonotoma*), there was no sufficient support for Pterostichini clades higher than the subgeneric level (Figure S3).

Unfortunately none of the five datasets revealed any information regarding the sister group of the *Ethira* clade. All of the Himalayan-Tibetan Pterostichini taxa included in our analyses that are outside the monophyletic *Ethira* clade cluster within different lineages of Pterostichini.

### Phylogeography of the Basal Lineages of the *Ethira* clade

Analyses of the combined Ribo + COI dataset (Figure 2), the 28S + 18S dataset, the 28S data alone (Figure S1), and the COI sequence data (Figure S4) each generate four basal lineages within the *Ethira* clade. These lineages are 1) the subgenus *Ethira* ( =  *Ethira* s.str.); 2) a clade comprised of *Pterostichus deuvei, P. gagates*, and *P. kleinfeldi*, which together form the morphological *gagates* group of the subgenus *Pseudethira*; 3) *P. kalo*, which is the only representative of the morphological *atrox* group included in our analysis; and 4) a clade designated “clade A”, which includes all of the remaining morphological species groups of the subgenus *Pseudethira* according to Schmidt [14]. Analysis of the 18S rDNA data alone resulted in no resolution within the *Ethira* clade, except for the *gagates* group (data not shown).

The answer to the question of what lineage represents the most basal branch of the *Ethira* clade differs among the analysed datasets. While previous morphological studies have provided evidence for sister group relationships with the subgenera *Ethira* and *Pseudethira* [14], the first split of the 28S tree produced in the present analyses separates the *gagates* group of *Pseudethira* (Figure S1). This result is corroborated by even higher Posterior Probability values in the combined Rib + COI analysis (Figure 2) and in the 28S + 18S analysis (data not shown). In contrast, the *atrox* group represents the first split of the COI tree (Figure S4).

**Figure 3:**
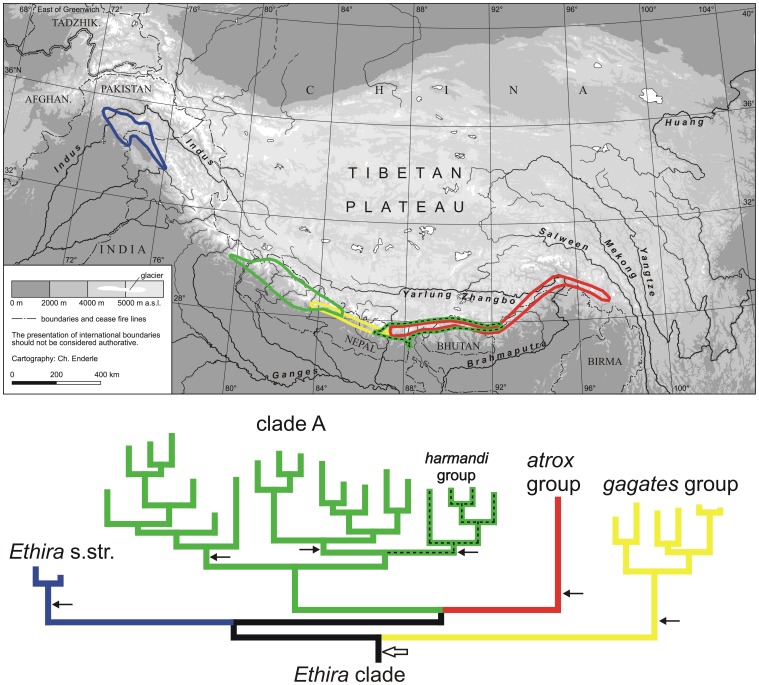
Comparison of phylogeny and distribution of the basal lineages of the *Ethira* clade. Below: The 50% majority rule consensus tree of the Bayesian analysis of the Rib + COI data set (see Figure 2). The four basal lineages are designated by different colors. The same colors are used to frame the distributional areas of each of the lineages in the map above the tree. The black arrows in the tree mark six events of wing reduction which necessitate from the assumption that the separated distributional area of the *harmandi* group originated from migration of a winged ancestor. The white arrow points to the single event of wing reduction that follows from the assumption of a Tibetan origin of the *Ethira* clade. For details see chapter discussion.

Each of the four basal lineages identified within the *Ethira* clade occurs in a restricted area along the High Himalayan mountain arc (Figure 3): *Ethira* s.str. is endemic to the Kashmir Himalaya; the *gagates* group is endemic to the Central Nepal Himalaya; the *atrox* group occurs only in the Eastern Himalaya; and clade A occurs in the Central and Eastern Himalaya, with a distributional gap being observed in eastern Central Nepal. As a caveat to our results, we note that we were only able to include in our analyses a single East Nepalese species from the *atrox* group, which includes a total of six species [31]. However, it is unlikely that the results will change if we include additional species in future analyses because this group is strongly supported by at least two relatively complex morphological synapomorphies (shortened mandibles and a reduction of elytral dorsal setation [30]). A similar conclusion can be reached regarding *Ethira* s.str. because this lineage is morphologically well defined by striking chaetotaxy characters of the head, pronotum, and elytra [31]. From *Ethira* s.str., we included three of the ten known taxa.

**Figure 4:**
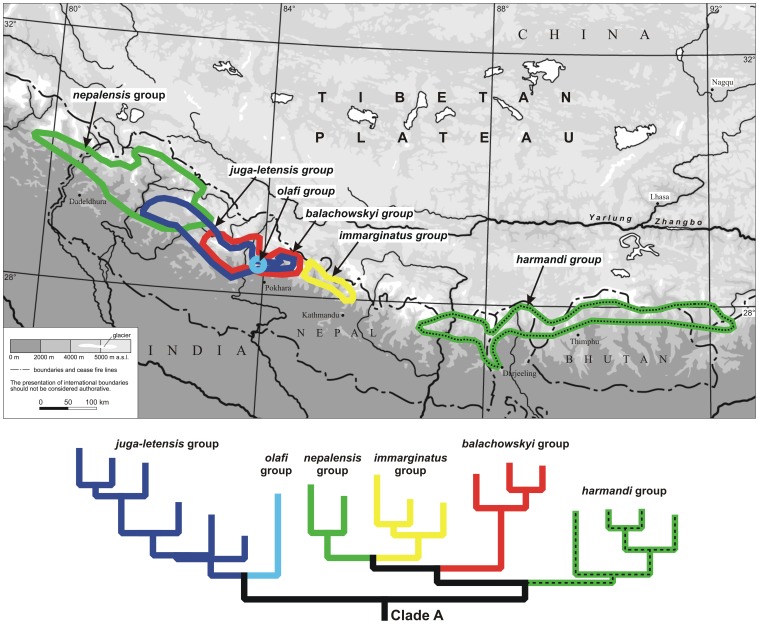
Comparison of phylogeny and distribution of the terminal lineages of sub-clade A of the *Ethira* clade as followed from the Bayesian analyses of the Rib + COI data set (see **Figure 2**).

Among all of the basal subclades of the *Ethira* clade, lineage A exhibits the largest species number and the widest distributional area. However, the eastern part of the disjunct distributional area of clade A, which extends along the High Himalaya from East Nepal in the West to the Indian province Arunachal Pradesh in the East, harbours species of only a single terminal lineage (*harmandi* group, Figures 3, 4). This lineage was likewise identified by a previous morphological study [14], [30]. All of the remaining species of clade A occur in the western part of the Central Himalaya (Figures 3, 4), and they do not represent a monophyletic group, as the East Himalayan *harmandi* group is identified as a sister group of a terminal lineage of clade A.

### Evidence for Distributional Gaps within the Range of the *Ethira* clade

The extensive distributional gap in the *Ethira* clade across the Kumaon Himalaya that divides *Ethira* s.str. geographically from all other species of the clade (Figure 3) is obvious in the combined Rib + COI data analyses (Figure 2), in the 28S + 18S data analyses (not shown) and in both the analyses of the 28S and COI sequence data alone (Figures S2, S3). Because in the West Nepal Himalaya only species of the terminal *nepalensis* group of clade A occur (Figure 4), the actual range of the gap extended eastwards at least to Central Nepal.

Additionally, in all these analyses, we found a distributional gap within clade A that is remarkable from a phylogeographic point of view. This gap divides the East Himalayan *harmandi* group from the rest of clade A, which is distributed in the western parts of the Central Himalaya (Figure 4). The gap stretches along the High Himalayan mountain arc over approx. 140 km from the Helambu-Langtang mountain range in the West to the southern slope of the Khumbu Himal in the east, and it therefore includes several separated High Himalayan mountain ranges. In this area, the polytypic species *P. gagates* of the monophyletic *gagates* group (Figure 2, Figures S2, S3) occurs as the only representative of the *Ethira* clade.

### Phylogeography of the Terminal Lineages of the *Ethira* clade

Differing from what was found for clade A, the species of the other more basal lineages of the *Ethira* clade are strictly allopatrically distributed. In contrast, in some areas of the Central Nepal Himalaya, up to four clade A species occur on the same mountain range. Moreover, clade A is by far the most species diverse. Therefore, the recent evolutionary history seems most enlightening with respect to clade A. We therefore concentrate on this lineage regarding the phylogeographic patterns of terminal species groups. The geographical distribution of the terminal lineages of clade A, which are supported by high Posterior Probabilities and/or high Bootstrap values in the combined Rib + COI data analysis is presented in Figure 4. A concentration of the lineages within the Central Nepal Himalaya is obvious, whereas only the *harmandi* group occurs in the eastern part, and only the *nepalensis* group is found in the western part. Although some doubts remain regarding the true relationships between the terminal lineages, mainly due to conflicts between the rDNA and mtDNA gene trees (see Figures S2 and S4), it can be stated with certainty that the eastern *harmandi* group and the western *nepalensis* group are both derived from ancestors who also have descendants in the Central Nepal Himalaya.

Regarding phylogeographic patterns at the species level, we concentrate on the results of the COI sequence analysis because the ribosomal genes show no or very low variability. The COI data strongly support local endemism of species and subspecies to separated slopes and valleys within the High Himalayan mountain range, as assumed by previous morphological studies. In addition, our analysis also identified several distinct lineages in cases where taxa do not show morphological variability between populations at different localities. For example, we observed deep branching between populations on different mountain ranges in the Central Nepalese species *P. kleinfeldi*, *P. juga*, and *P. chainapaani* (Figures S3, S4). Moreover, the geographical separation of the COI sequences along the southern slopes of the High Himalayan mountain arc is so markedly developed that the geographical origin of each population can be determined in all cases. In contrast, the variability of the COI sequences is very low or nonexistent within populations.

**Figure 5:**
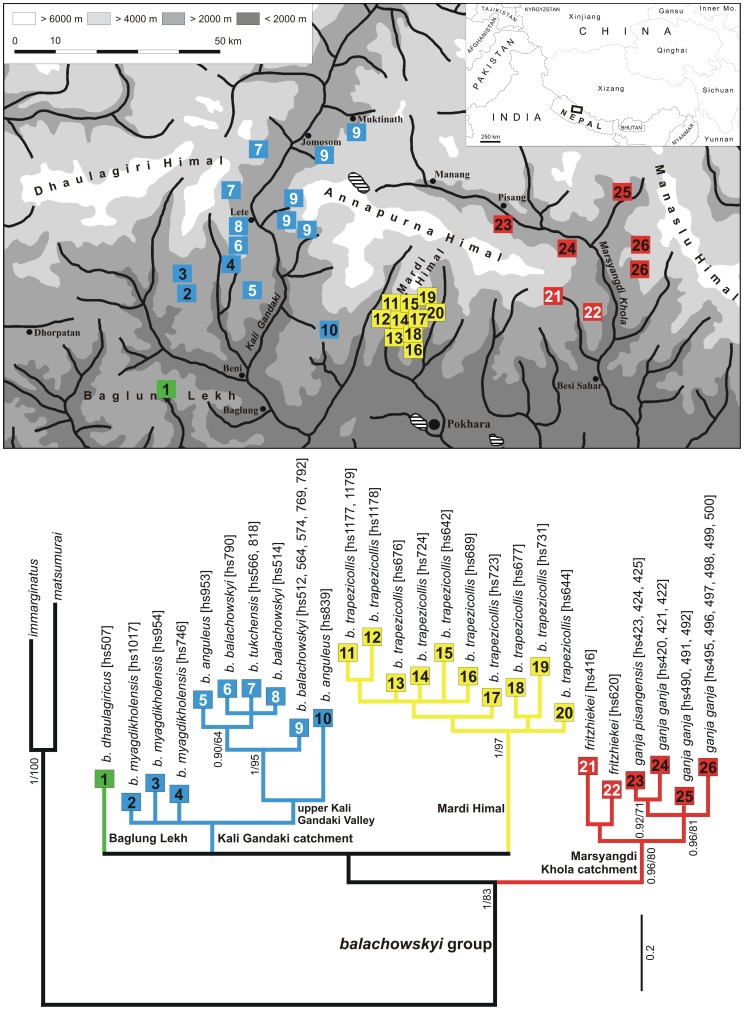
Comparison of mitochondrial phylogeny and geographical distribution of the *balachowskyi* species group. Below: The 50% majority rule consensus tree of the Bayesian analysis of the COI sequences using *Pterostichus immarginatus and P. matsumurai* as outgroups. Numbers on or beside branches are Posterior Probabilities ≥90%/Bootstrap support values. Numbers in brackets refer to the internal specimen identification code. Private haplotypes are numbered in colored boxes. The collecting localities of these haplotypes are shown in the map above.

For example, the phylogeographic patterns of the terminal *balachowskyi* group of clade A, which is monophyletic in all molecular data analyses as well as in a previous morphological analysis [14], are shown in Figure 5. The deepest branches of this group divide populations from different valley systems. Within these branches, low but significant sequence divergence divides all of the populations on southern High Himalayan slopes that are geographically separated by deep gorges and south stretching mountain ridges. We were able to detect slightly extended distributional areas of haplotypes only along transverse valleys of the Inner Himalaya. Similar phylogeographic patterns are observed to have developed in all of the other terminal lineages of the *Ethira* clade.

### Divergence Time Estimations within the *Ethira* clade

As noted above, the divergence time estimations for the *Ethira* clade must be considered with caution and can only be viewed as an initial rough approximation because the basis for fossil calibration is still highly insufficient. This is documented by the fact that the confidence intervals resulting from the BEAST analysis were very large (Figure S5). Nevertheless, all calibration schemes produced comparable results with the exception of using mutation rates alone (see Table S4). Most importantly, both calibration points produced similar results regarding the dating of the *Ethira* clade with the mean node age of the first branching within the clade estimated to be around 18–20 million years. Also, both calibration points when used independently, derived plausible ranges for the respective other calibration point. Only the calibration scheme based on published mutation rates alone produces entirely different age estimations with a mean of the *Ethira* clade estimated to be 5.7 MY (Table S4). It is noteworthy that this scheme likewise produces very low age-estimates for the Euro-Alpine clade (2.8 MY) as well as *Pterostichus* clade (10.7 MY). The mean substitution rates for Pterostichini derived from the different calibration schemes range from 0.0031–0.0039 substitutions/site/MY in the schemes including calibration points and 0.019 substitutions/site/MY when using the published rates as a prior alone. The range of the ucld.stdev parameter between 0.47 and 0.64 (see Table S4) indicated a rather stable molecular clock throughout the tree.

According to calibration point estimation the primary diversification of the clade into the four basal lineages (*Ethira* s.str., *atrox* group, *gagates* group, and clade A) was completed prior to 8–19 million years ago, as shown by the first detected bifurcation within clade A (estimated node age 13 MY, Figure S5). For the subsequent bifurcations no evidence for periods of higher or lower diversification rates in clade A and in the *gagates* group up to the present exists (the relevant conditions are unknown for *Ethira* s.str. and the *atrox* group, as an insufficient number of species were included in the analyses). It is not unlikely that the evolution of some species could extend back to the Upper Miocene, such as for *P. deuvei* and *P. kleinfeldi* of the *gagates* group. For these species, we obtained node estimations of 3–9 MY. However, some pairs of allopatric species are of relatively recent origin, such as *P. angoarnigi + P. thanglaensis* (0.1–1.5 MY), *P. gompanus* + *P. tulobalu* (0.1–2.7 MY), and *P. jaljaleensis + P. pseudoharmandi* (0.1–1.4 MY).

## Discussion

### Evidence for the Primary Origin of the *Ethira* clade

The phylogenetic analyses convincingly demonstrate the monophyly of the *Ethira* clade. Furthermore, the analyses support our hypothesis that the *Ethira* clade has a long evolutionary history in the southern part of the Himalayan-Tibetan mountain system, which goes back to the Miocene according to our data, though this dating must still be considered cautiously at this point. Unfortunately the in this context important question of the sister group of the *Ethira* clade is still unresolved. It seems likely that the sister taxon was not part of our sampling, despite the very comprehensive 28S dataset analysed in this study. However, given that we sampled all of the relevant Pterostichini species groups, as discussed in the Materials and Methods, our analyses do reveal that the sister taxon of the *Ethira* clade does not occur in the Himalaya and very likely also does not inhabit the mountains adjacent to the High Himalayan mountain arc. Immigration from adjacent lowlands and hills of the Indian subcontinent is also highly improbable as the genus *Pterostichus* is completely absent in the tropics. In fact, the results of the phylogenetic analyses lead to the assumption that the *Ethira* ancestor did not originate from immediately adjacent parts of South and Central Asia but from the temperate zone of the Miocene Boreal. The *Ethira* clade could have evolved from a winged ancestor that inhabited the southern margin of the Himalaya-Tibet orogen during an early phase of the uplift when cloud forests on this part of the mountain system were geographically separated from similar habitats in southern Central Asia. This is significant considering that during the early uplift history, the southern edge of the orogen was situated at lower latitudes than today [52].

Our divergence time estimations are still provisional as more calibration points are needed in the future. However, the two calibration points available, produce plausible results given that they correctly crossdate each other and that the derived mutation rates are well in the range of published mutation rates [46], [47], [53], [54] with the exception of the very fast rates derived for *Trechus* on the Canary Islands [49]. As the calibration scheme based on this rate produced an unrealistically young age estimate for the *Pterostichus* clade (see Baltic Amber fossils, mentioned above), these results can be dismissed. Thus, our dating places the immigration of the *Ethira* ancestor into the Himalaya-Tibet orogen between the Lower and the Middle Miocene. More precise dating of the immigration history and reaching more concrete conclusions regarding from which part of the Miocene Boreal the *Ethira* clade ancestor migrated into the Himalayan-Tibetan mountain system will only be possible once the recent sister group is known. For this to be determined, a substantial expansion of sampling including extra-Himalayan taxa will be necessary in the future.

### Evidence for the Genesis of the Distributional Area of the *Ethira* clade

In attempting to understand the distributional history of the *Ethira* clade, it seems most noteworthy that there is no evidence from our analyses that modern species are able to migrate across separated mountain ranges and valley systems paralleling the High Himalayan mountain arc to any significant degree. Rather, the separation of the populations seems highly effective because in each of the investigated species, the distribution of the private COI haplotypes along the W-E extension of the Himalaya was found to be extremely limited. Findings of identical haplotypes in adjacent side valleys provided evidence of dispersal in recent times, solely from populations occurring in the upper parts of Himalayan transverse valleys where the valley bottoms lie higher than 2000 m above sea level and are therefore situated well above the tropical zone (Figure 5). Against this background, we conclude that the restricted distribution of all of the identified natural species groups of the *Ethira* clade results from their ancestors exhibiting the same strongly limited dispersal ability. The richness of lineages in the Central Himalaya can also be explained by this argument, given that this area was actually part of the ancestral distributional area. However, the wide distribution of the *Ethira* clade across the entire Himalayan mountain arc and the large distributional gaps observed (Figures 3, 4) both necessitate the assumption that dispersal options along the southern part of the Himalayan-Tibetan orogen were better during previous periods in the evolutionary history of these taxa. There are two reasonable alternatives to explain such improved dispersion: either the ancestral species of the respective lineages were able to fly, or a different topography at the time allowed much easier dispersion compared to the recent past even for wingless ground beetles.

These alternatives lead to contradictory theories of Himalayan faunal genesis. The immigration hypothesis implies that the ancestral species of the modern lineages had the ability to fly, as colonisation of the High Himalayan mountain arc throughout its entire extent by wingless high altitude ground beetles coming from their postulated centres of origin in regions to the Northwest or the East of the Himalaya is practically impossible. This also holds true for phases of limited vertical extension of the Himalaya, as this mountain arc has always been interspersed with epigenetic transverse valleys that form effective barriers to dispersal. The results of the present phylogeographic study indicate that the immigration hypothesis would necessitate the existence of winged species very late in the phylogeny of the *Ethira* clade, at least until the separation of the *harmandi* group from clade A, independent of whether immigration of the *Ethira* clade ancestor occurred from the northwest or from the east into the Himalaya. Consequently, we have to assume that the evolution of winglessness with comprehensive deformations of the exoskeleton occurred independently six times in the evolutionary history of the *Ethira* clade (Figure 3). Because migration paralleling the High Himalayan mountain arc necessitates stepwise colonisation of separate mountain ranges, we have to further assume that two events of large-scale extinction occurred, one across the Kumaon Himalaya, separating *Ethira* s.str. by distance of more than 500 km from all other lineages of the clade, and one across eastern Central Nepal, which separated the *harmandi* group from clade A by a distance of approx. 140 km. However, due to the lack of evidence for climatic or other abiotic anomalies in the relevant areas, it seems impossible to explain such large-scale extinctions. In the case of the separation of the *harmandi* group, it is noteworthy that the distributional gap associated with clade A is part of the distributional area of the *gagates* group. Species of this group show no markedly different ecological preferences compared to species of clade A, which follows from syntopical occurrences of species of both these lineages in other parts of Central and East Nepal. This observation based on field research emphasises the difficulties of interpreting distributional gaps ecologically.

In contrast, the hypothesis of an origin of Himalayan faunal elements with a low dispersal ability in South Tibet, *sensu* Schmidt [14] and Schmidt & Hartmann [15], assumes a single loss of the ability to fly during the earliest phase in the evolution of the ground beetle lineages when relevant ancestors had adapted to the high altitude environment in South Tibet, prior to the final uplift of the High Himalaya. Thus, winglessness and corresponding deformations of the exoskeleton could have evolved only once in the *Ethira* clade, before the first cladogenesis (Figure 3). In addition, only one large-scale extinction event is needed to explain both of the extended distributional gaps described above. However, this extinction must have been a long process and must have affected the entire primary distributional area of the *Ethira* clade. This scenario completely agrees with what is known from the climatic history of the area (see Introduction), as South Tibet was continuously drying in the course of the subsequent uplift of the High Himalayan mountain belt. This mountain belt ultimately acts as an effective barrier to the northward streaming humid air masses associated with the Indian summer monsoons [55]. The last piece of evidence for the existence of cloud forests in the Tibetan Himalaya north of Central Nepal comes from fossil data and dates back to the Late Pliocene [56], [57].

When primary diversification within the *Ethira* clade occurred in the high altitudes of South Tibet prior to the uplift of the High Himalaya, the strong restriction detected with respect to the modern geographical distribution of each of the terminal lineages in the High Himalaya became apparent. The ancestral species of these lineages were forced to follow the mountain slopes paralleling the epigenetic transverse valleys in the course of the rise and growth of the High Himalaya, which otherwise formed an effective barrier against the North-South dispersal of these species. The fact that dispersal of wingless ground beetles along transverse valleys is generally more successful than across valleys and mountain ridges also becomes apparent from the data analysed in the present study (Figure 5). The large distributional gaps we were able to identify between some of the *Ethira* clade lineages in our phylogeographic analyses and that are known to occur among several other wingless ground beetle taxa of the Himalaya are simple to explain due to the legitimate assumption that a North-South shift of the distribution of these species was not possible at every site along the mountain belt or for each of the lineages that had evolved in South Tibet. In contrast, if one considers the particular geo-morphological situation along the southern margin of the Tibetan Plateau, distributional gaps would be expected to be the rule, rather than an exception for all of those taxa having an origin in South Tibet. Finally, if we consider these last arguments, it is no more surprising that *Ethira* s.str. is by far the most separated lineage geographically. This lineage evolved on the western macro-slope of the Himalaya-Tibetan orogen, which is drained by the Indus catchment. Like all of the other main drainage systems of the southern part of the Himalaya Tibetan mountain system, the Indus River originates from the central part of the orogen (Figure 1). In the course of the stepwise uplift and drying of Tibet, the wingless ancestors of the modern *Ethira* s.str. species were only able to migrate along the slopes of the Tibetan Himalaya and the Transhimalaya paralleling the upper Indus valley more than 500 km to the northwest until reaching the transverse Indus valley in the Kashmir Himalaya. The southwestern slopes of the Kashmir Himalaya are the distributional area of the modern species. Our divergence time estimation indicates a time range of 6–15 mya in which this considerable range shift could have occurred along the upper Indus Valley (Figure S5). Considering the migration abilities of wingless ground beetle species and the given geomorpholocical setting, we find this to be a reasonable estimate.

### Conclusions

The molecular genetic approach used in this study provides several pieces of evidence that strongly support an origin of High Himalayan forest dwelling faunal elements with a low dispersal ability in South Tibet. The Tibetan origin hypothesis explains the evolution, diversity and distribution of the Himalayan ground beetle *Ethira* clade much more parsimoniously than the alternative immigration hypothesis. The Tibetan origin hypothesis succeeds in explaining apparently paradoxical distributional patterns detected in our phylogeographic analyses, such as the Central Himalayan endemism of highly diverse species groups and the wide distributional gaps between related lineages of wingless ground beetles. Furthermore, the Tibetan origin hypothesis agrees with modern geological scenarios, which assume that primary uplift occurred in the southern parts of the Tibetan Plateau [58], [59], and with fossil evidence of Pliocene cloud forests on the northern slope of the Himalaya in South Tibet [56], [57]. In contrast, the immigration hypothesis, which assumes that the high montane forest fauna originated in Western Asia, Eastern Tibet, and the mountains of Indochina and, thus, postulates that subsequent migration of the ancestors took place along the High Himalayan mountain arc, requires several additional assumptions to explain the modern distributional patterns of wingless ground beetle taxa. The most obvious weakness of the immigration hypothesis lies in the severe difficulty of explaining the wide separation of species groups endemic to the High Himalaya, and the large distributional gaps between lineages of high altitude ground beetles. Under the framework of the immigration hypothesis, there are no known ecological factors (extant or past) that could explain complete subsequent extinctions in mountains east and northwest of the Himalaya as well as in vast areas within the Himalayan mountain belt.

Given the apparently contrasting immigration patterns found among taxonomic groups with a strong dispersal ability (e.g. [4], [10], [11]), the Tibetan origin hypothesis seems to be a more suitable hypothesis for explaining the evolutionary and biogeographical history of taxonomic groups with low dispersal abilities. However, against the background of a high probability of Miocene-Pliocene forests existing in South Tibet and considering the results of biogeographical explorations of Himalayan Tibetan birds performed by Weigold [12], it seems very likely that the Tibetan origin hypothesis could also be a suitable instrument for explaining modern distributional patterns in some Himalayan species groups with a strong dispersal ability. We consider it most likely that a Tibetan origin hypothesis exists in many groups, but that its patterns have been superimposed on more recent colonisation events, especially in groups with stronger dispersal abilities. Thus, we hope that our study provokes additional analyses including a range of additional taxonomic groups.

## Supporting Information

Figure S1 **The 50% majority rule consensus tree of the Bayesian analyses of the 28S rDNA sequence data of Pterostichini (** ***Ethira*** **clade, 43 additional** ***Pterostichus*** **subgeneric taxa, and 13 putatively related genera) using** ***Abax parallelepipedus*** **and** ***Molops spartanus*** **as outgroup taxa.** Species and species groups known to occur in the Himalayan-Tibetan mountain system are marked with an asterisk. Numbers on or beside branches are Posterior Probabilities ≥90%/Bootstrap support values. Numbers in brackets refer to the GenBank accession number and to the internal specimen identification code, respectively, and are added in cases when more than one specimen of a morphological species was added to the analysis. The subtree of the *Ethira* clade is shown in Figure S2. Abbreviation: *P.  =  Pterostichus*.
(TIF)

Figure S2 ***Ethira*** **clade subtree of the Bayesian analyses of the 28S rDNA sequence data of Pterostichini shown in. Figure S1.** Numbers on or beside branches are Posterior Probabilities ≥90%/Bootstrap support values. Numbers in brackets refer to the internal specimen identification code and are added in cases when more than one specimen of a morphological species was added to the analysis. Beside the four basal lineages of the *Ethira* clade six of the terminal lineages of clade A are designated which are discussed in the text (the paraphyletic *immarginatus* group in this tree is highly supported by the analyses of the Rib + COI data set and of the COI data, see text Figure 2 and Figure S4). Abbreviations: *P.  =  Pterostichus; Ps.  =  Pseudethira*.
(TIF)

Figure S3 **The 50% majority rule consensus tree of the Bayesian analyses of the COI mtDNA sequence data of Pterostichini (** ***Ethira*** **clade, 11 additional** ***Pterostichus*** **subgeneric taxa, and 7 putatively related genera) using** ***Abax parallelepipedus*** **as outgroup taxon.** Numbers on or beside branches are Posterior Probabilities ≥90%/Bootstrap support values. Numbers in brackets refer to the internal specimen identification code and are added in cases when more than one specimen of a morphological species was added to the analysis. The clade A sub-tree of *Ethira* is shown in Figure S4. Abbreviations: *P.  =  Pterostichus; Ps.  =  Pseudethira*.
(TIF)

Figure S4 **Clade A subtree of the Bayesian analyses of the COI rDNA sequence data set shown in Figure S3.** Numbers on or beside branches are Posterior Probabilities ≥90%/Bootstrap support values. Numbers in brackets refer to the internal specimen identification code and are added in cases when more than one specimen of a morphological species was added to the analysis. Six of the terminal species groups are designated and are discussed in the text (the paraphyletic *harmandi* group and the polyphyletic *juga-letensis* group in this tree are highly supported by the Rib + COI data analysis). Branching patterns of the *balachowskyi* group are shown in text Figure 5. Abbreviations: *P.  =  Pterostichus; Ps.  =  Pseudethira*.
(TIF)

Figure S5 **Chronogram of** ***Pterostichus*** **haplotypes based on the COI data set inferred from a Bayesian analysis with BEAST.** Bars on nodes represent 95% confidence intervals of node ages (see text for further details).
(TIF)

Table S1 **Specimen identification codes.** If the material was not obtained from GenBank the following resources were used to identify specimens (marked in the list by a superscripted number at the end of species citation): 1[63]; 2[64]; 3[65]; 4[66]; 5[31]; 6[14]; 7[67]; 8[30]; 9[62]; 10[68]; 11[69]; 12[60]; 13[61]; 14[70]; 15 unpublished data from revisional works of D.W. Wrase (Berlin) and J. Schmidt (Marburg and Rostock). Names with species epithet replaced by “spec.” indicate that identification was impossible due to the lack of species group revisions. Names prefaced with “cf.” indicate that it is unclear whether or not the specimen belongs to that species. The internal code is used to associate DNA sequence data and voucher specimens and is included in the tree diagrams for species or subspecies represented by multiple individuals.
(DOCX)

Table S2 **Primers used for DNA amplification (amp) and sequencing (seq).**
(DOCX)

Table S3 **PCR thermal cycling condition used.**
(DOCX)

Table S4 **Divergence time estimates for the** ***Ethira*** **clade, the Euro-Alpine** ***Pterostichus*** **clade, and for the genus** ***Pterostichus*** **from five different calibration schemes (details see main text).**
(DOCX)
