# Supplementary figures and images for: Into the Himalayan Exile: The Phylogeography of the Ground Beetle Ethira clade Supports the Tibetan Origin of Forest-Dwelling Himalayan Species Groups
Source: PLoS One. 2012 Sep 26;7(9):e45482. doi: 10.1371/journal.pone.0045482 (PMC3458877; doi:10.1371/journal.pone.0045482)

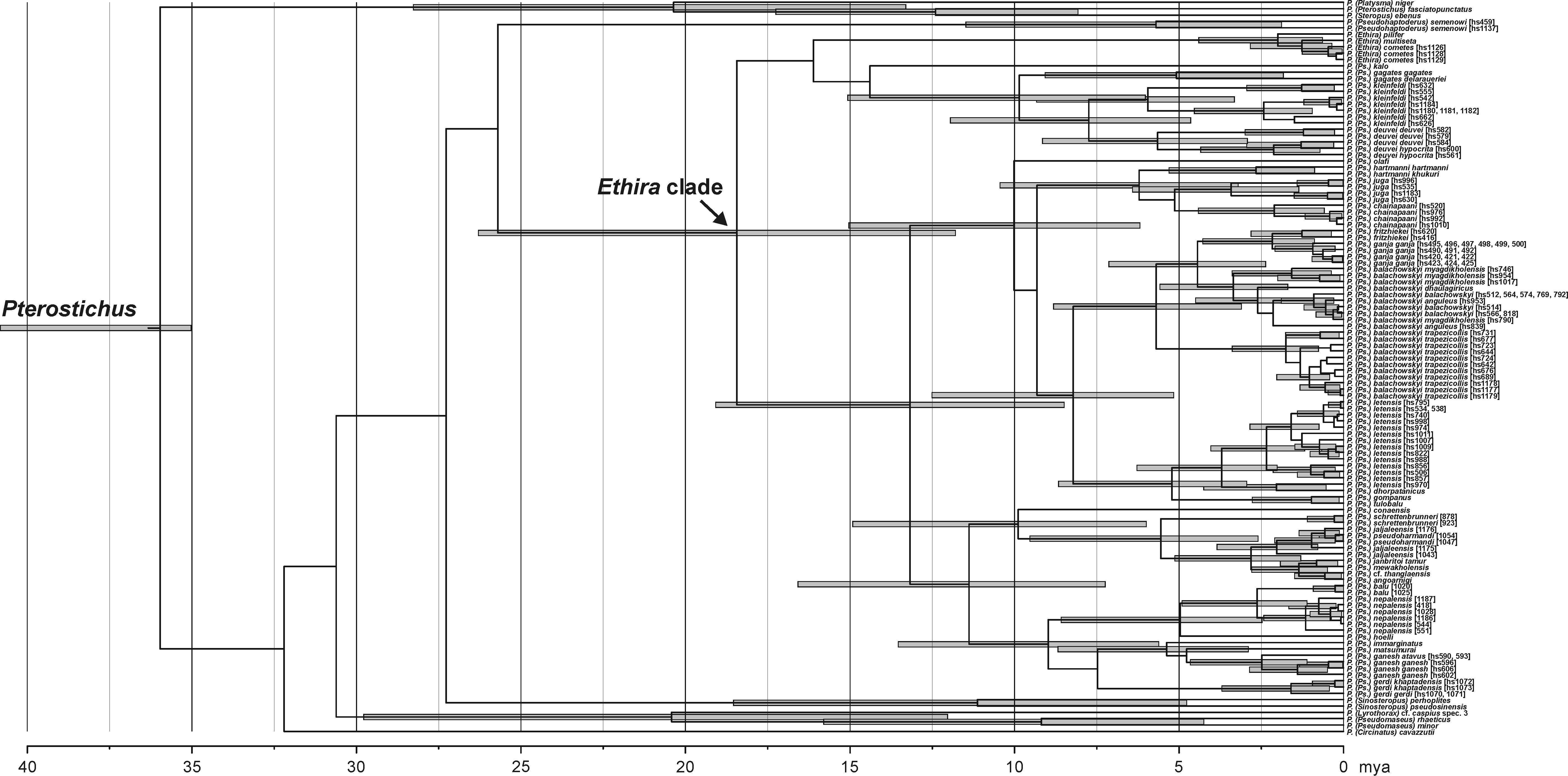

Supplement: Figure S5 — Chronogram of Pterostichus haplotypes based on the COI data set inferred from a Bayesian analysis with BEAST. Bars on nodes represent 95% confidence intervals of node ages (see text for further details). (TIF) [file pone.0045482.s005.tif]
