# Supplementary material for: Into the Himalayan Exile: The Phylogeography of the Ground Beetle Ethira clade Supports the Tibetan Origin of Forest-Dwelling Himalayan Species Groups
Source: PLoS One. 2012 Sep 26;7(9):e45482. doi: 10.1371/journal.pone.0045482 (PMC3458877; doi:10.1371/journal.pone.0045482)
Supplement: Table S1 — Specimen identification codes. If the material was not obtained from GenBank the following resources were used to identify specimens (marked in the list by a superscripted number at the end of species citation): 1[63]; 2[64]; 3[65]; 4[66]; 5[31]; 6[14]; 7[67]; 8[30]; 9[62]; 10[68]; 11[69]; 12[60]; 13[61]; 14[70]; 15 unpublished data from revisional works of D.W. Wrase (Berlin) and J. Schmidt (Marburg and Rostock). Names with species epithet replaced by “spec.” indicate that identification was impossible due to the lack of species group revisions. Names prefaced with “cf.” indicate that it is unclear whether or not the specimen belongs to that species. The internal code is used to associate DNA sequence data and voucher specimens and is included in the tree diagrams for species or subspecies represented by multiple individuals. (DOCX) [file pone.0045482.s006.docx]

**Table S1** Specimen identification codes. If the material was not obtained from GenBank the following resources were used to identify specimens (marked in the list by a superscripted number at the end of species citation): ^1^[64]; ^2^[65]; ^3^[66]; ^4^[67]; ^5^[32]; ^6^[14]; ^7^[68]; ^8^[31]; ^9^[63]; ^10^[69]; ^11^[70]; ^12^[61]; ^13^[62]; ^14^[71]; ^15^unpublished data from revisional works of D.W. Wrase (Berlin) and J. Schmidt (Marburg and Rostock). Names with species epithet replaced by “spec.” indicate that identification was impossible due to the lack of species group revisions. Names prefaced with “cf.” indicate that it is unclear whether or not the specimen belongs to that species. The internal code is used to associate DNA sequence data and voucher specimens and is included in the tree diagrams for species or subspecies represented by multiple individuals.

| **Species/subspecies** | Origin | **Latitude** | **Date** | Genbank and internal codes (hs) | | |
| --- | --- | --- | --- | --- | --- | --- |
|  |  | **Longitude** | **Collector** | **COI** | 28S | 18S |
| *Abax parallelepipedus* (Piller & Mitterpacher, 1783)^1^ | GERMANY: Mecklenburg, Ehbruch, alt. 3 m | 54°09’  11°58’ | 24.IX.2009  J. Schmidt | hs1145 | hs1145 | hs1145 |
| *Agonum extensicolle* (Say, 1823) | GenBank | na | na | na | na | AF002775 |
| *Agonum muelleri* (Herbst, 1784) | GenBank | na | na | na | na | FJ173119 |
| *Amara aenea* (DeGeer, 1774) | GenBank | na | na | na | na | FJ173123 |
| *Aristochroa* spec. | CHINA: SE Tibet, E-slope Mila Pass, near Songduo, alt. 4000-4400m | na | 11.VII.2009  M.-Y. Tian | hs1133 | na | na |
| *Bembidion chalceum* Dejean, 1831 | GenBank | na | na | na | na | gi149347430 |
| *Chlaenius ruficauda* Chaudoir, 1856 | GenBank | na | na | na | na | AF012473 |
| *Chlaenius vestitus* (Paykull, 1790) | GenBank | na | na | na | na | AF201404 |
| *Cyclotrachelus ovulum* (Chaudoir, 1868) | GenBank | na | na | na | EU142327 | na |
| *Cymindis punctigera* (LeConte, 1851) | GenBank | na | na | na | na | AF002773 |
| *Cymindis punctigera* (LeConte, 1851) | GenBank | na | na | na | na | AF002773 |
| *Discoderus cordicollis* (G. Horn, 1891) | GenBank | na | na | na | na | AF012472 |
| *Harpalus affinis* (Schrank, 1781) | GenBank | na | na | na | na | gi219878575 |
| *Lesticus holzschuhi* Straneo, 1985^2^ | NEPAL: N of Pokhara, above Lahachok, alt. 2000 m | 28°20’  83°55’ | 15.V.2008  J. Schmidt | hs909 | hs909 | hs909 |
| *Lesticus magnus* (Motschulsky, 1860) | GenBank | na | na | na | AB243465 | na |
| *Lophoglossus haldemanni* (LeConte, 1848) | GenBank | na | na | na | EU142437 | na |
| *Molops spartanus* (Schaum, 1862) | GenBank | na | na | na | AB243477 | na |
| *Myas chalybeus* (Palliardi, 1825) | GenBank | na | na | na | AB243478 | na |
| *Myas coracinus* (Say, 1823) | GenBank | na | na | na | EU142438 | na |
| *Nirmala indica* Hope, 1831^15^ | NEPAL: Kathmandu, Shivapuri Danda, S Okreini, alt. 2100-2200 m | 27°47’  85°24’ | 22.V.2005  J.Schmidt | hs908 | na | na |
| *Nirmala odelli* Andrewes, 1930^15^ | NEPAL: Taplejung Distr., Milke Danda, alt. 3000 m | na | 16.VI.2009  P. Rai | na | hs1051 | hs1051 |
| *Paranchus albipes* (Fabricius, 1796) | GenBank | na | na | na | na | AF201403 |
| *Piesmus submarginatus* (Say, 1823) | GenBank | na | na | na | EU142439 | na |
| *Poecilus cupreus* (Linnaeus, 1758) | GenBank | na | na | DQ295309 | na | na |
| *Poecilus lucublandus* (Say, 1823) | GenBank | na | na | na | EU142440 | EU142291 |
| *Poecilus samurai* (Lutshnik, 1916) | GenBank | na | na | na | AB243500 | na |
| *Poecilus versicolor* (Sturm, 1824) | GenBank | na | na | na | AB243479 | na |
| *Pterostichus* (*Abacidius*) *atratus* (Newman, 1838) | GenBank | na | na | na | EU142441 | na |
| *P.* (*Abea*) *yamauchii* Morita, 1992 | GenBank | na | na | na | AB243459 | na |
| *P.* (*Anilloferonia*) *testaceus* (Van Dyke, 1926) | GenBank | na | na | na | EU142326 | na |
| *P.* (*Argutor*) *dulcis* (Bates, 1883) | GenBank | na | na | na | AB243463 | na |
| *P.* (*Argutor*) *sulcitarsis* A. Morawitz, 1862 | GenBank | na | na | na | AB243473 | na |
| *P.* (*Badistrinus*) *bandotaro* Tanaka, 1958 | GenBank | na | na | na | AB243462 | na |
| *P.* (*Bothriopterus*) *abasarukini* O. & E. Berlov, 1996 | GenBank | na | na | na | AB243489 | na |
| *P.* (*Bothriopterus*) *adstrictus* Eschscholtz, 1823 | GenBank | na | na | na | AB243497 | na |
| *P.* (*Bothriopterus*) *aeneocupreus* (Fairmaire, 1887)^3^ | CHINA: South Tibet, Reting Tsangpo valley, alt. 4750 m | 30°24’48  91°41’41 | 18.VII.2007  J. Schmidt | hs461 | hs461 | hs461 |
| *P.* (*Bothriopterus*) *aeneocupreus* (Fairmaire, 1887)^3^ | NEPAL: Manang Distr., Thorung Phedi, alt. 4500 m | 28°47’  83°59’ | 14.V.2007  J. Schmidt | hs572 | hs572 | na |
| *P.* (*Bothriopterus*) *aeneocupreus* (Fairmaire, 1887)^3^ | NEPAL: Manang Distr., Thorung Phedi, alt. 4500 m | 28°47’  83°59’ | 14.V.2007  J. Schmidt | na | hs573 | na |
| *P.* (*Bothriopterus*) *mutus* (Say, 1723) | GenBank | na | na | na | EU142443 | na |
| *P.* (*Bothriopterus*) *oblongopunctatus* (Fabricius, 1787) | GenBank | na | na | na | AB243480 | na |
| *P.* (*Bothriopterus*) *subovatus* (Motschulsky, 1860) | GenBank | na | na | na | AB243474 | na |
| *P.* (*Circinatus*) *cavazzutii* Allegro & Sciaky, 2010^4^ | CHINA : Sichuan Prov., Lizi Ping, alt. 2750 m | 28°58'  102°25' | 26.V.2011  J. Martens | na | hs1189 | na |
| *P.* (*Circinatus*) *cavazzutii* Allegro & Sciaky, 2010^4^ | CHINA : Sichuan Prov., Lizi Ping, alt. 2750 m | 28°58'  102°25' | 26.V.2011  J. Martens | na | hs1190 | na |
| *P.* (*Circinatus*) *cavazzutii* Allegro & Sciaky, 2010^4^ | CHINA : Sichuan Prov., Lizi Ping, alt. 2750 m | 28°58'  102°25' | 26.V.2011  J. Martens | hs1191 | hs1191 | hs1191 |
| *P.* (*Cryobius*) *kurosawai* Tanaka, 1958 | GenBank | na | na | na | AB243485 | na |
| *P.* (*Cryobius*) *riparius* (Dejean, 1828) | GenBank | na | na | na | EU142445 | na |
| *P.* (*Cyclotrachelus*) *ovulum* (Chaudoir, 1868) | GenBank | na | na | na | EU142327 | na |
| *P.* (*Cylindrocharis*) *rostratus* (Newman, 1838) | GenBank | na | na | na | EU142446 | na |
| *P.* (*Eosteropus*) *alacer* A. Morawitz, 1862 | GenBank | na | na | na | AB243488 | na |
| *P.* (*Eosteropus*) *karasawai* Tanaka, 1958 | GenBank | na | na | na | AB243452 | na |
| *P.* (*Eosteropus*) *moestus* (Say, 1823) | GenBank | na | na | na | EU142447 | na |
| *P.* (*Eosteropus*) *orientalis* (Motschulsky, 1844) | GenBank | na | na | na | AB243453 | na |
| *P.* (*Ethira*) *cometes* (Andrewes, 1936)^5^ | INDIA, Jammu & Kashmir, Poonch Distr., Loran, alt. 2300 m | na | 17.VIII.2010  C. Reuter | hs1126 | hs1126 | hs1126 |
| *P.* (*Ethira*) *cometes* (Andrewes, 1936)^5^ | INDIA, Jammu & Kashmir, Poonch Distr., Loran, alt. 2300 m | na | 17.VIII.2010  C. Reuter | hs1128 | na | na |
| *P.* (*Ethira*) *cometes* (Andrewes, 1936)^5^ | INDIA, Jammu & Kashmir, Poonch Distr., Loran, alt. 2300 m | na | 17.VIII.2010  C. Reuter | hs1129 | na | na |
| *P.* (*Ethira*) *multiseta* (Straneo, 1984)^5^ | PAKISTAN, North West Frontier Province, Shogran, Malkandi, alt. 3050 m | na | 17.VI.1997  K. Staven | hs931 | na | na |
| *P.* (*Ethira*) *pilifer* (Bates, 1878)^5^ | PAKISTAN: North West Frontier Province, Nathiagali, alt. 1200 m | 34°40’  73°24’ | 26.IV.2009  C. Reuter | hs939 | hs939 | hs939 |
| *P.* (*Eurythoracana*) *kajimurai* Habu & Tanaka, 1957 | GenBank | na | na | na | AB243498 | na |
| *P.* (*Feronina*) *palmi* Schaeffer, 1910 | GenBank | na | na | na | EU142449 | na |
| *P.* (*Gastrosticta*) *tumescens* LeConte, 1863 | GenBank | na | na | na | EU142450 | na |
| *P.* (*Georgeballius*) *hoplites* Bates, 1883 | GenBank | na | na | na | EU142451 | na |
| *P.* (*Hypherpes*) *lama* Ménétries, 1843 | GenBank | na | na | na | EU142370 | na |
| *P.* (*Hypherpes*) *menetriesii* LeConte, 1873 | GenBank | na | na | na | EU142372 | EU142282 |
| *P.* (*Hypherpes*) *serripes* (LeConte, 1875) | GenBank | na | na | na | EU142382 | na |
| *P.* (*Hypherpes*) *tarsalis* LeConte, 1873 | GenBank | na | na | na | EU142389 | na |
| *P.* (*Japeris*) *defossus* Bates, 1883 | GenBank | na | na | na | AB243460 | na |
| *P.* (*Lamenius*) *caudicalis* (Say, 1823) | GenBank | na | na | na | EU142452 |  |
| *P.* (*Lenapterus*) *subrugosus* Straneo, 1955 | GenBank | na | na | na | AB243484 | na |
| *P.* (*Leptoferonia*) *lobatus* Hacker, 1968 | GenBank | na | na | na | EU142427 | na |
| *P.* (*Leptoferonia*) *marinensis* Hacker, 1968 | GenBank | na | na | na | EU142428 | na |
| *P.* (*Leptoferonia*) *pemphredo* Will, 2007 | GenBank | na | na | na | EU142431 | na |
| *P.* (*Leptoferonia*) *sphodrinus* LeConte, 1863 | GenBank | na | na | na | EU142433 | na |
| *P.* (*Lianoe*) *mirificus* Bates, 1883 | GenBank | na | na | na | AB243449 | na |
| *P.* (*Lyrothorax*) cf. *caspius* (Ménéntriés, 1832) spec. 1 | GenBank | na | na | na | AB243469 | na |
| *P.* (*Lyrothorax*) cf. *caspius* (Ménéntriés, 1832) spec. 2^15^ | IRAN: Mazandaran Prov., vic. Now Shar, alt. 880 m | 36°31'  51°38' | 2.V.2010  A. Weigel | na | hs1138 | hs1138 |
| *P.* (*Lyrothorax*) cf. *caspius* (Ménéntriés, 1832) spec. 3^15^ | IRAN: Mazandaran Prov., vic. Kolijak, alt. 1850 m | 36°28'  51°39' | 5.V.2010  A. Weigel | hs1139 | hs1139 | na |
| *P.* (*Lyrothorax*) *fujitai*  Tanaka & Ishida, 1972 | GenBank | na | na | na | AB243483 | na |
| *P.* (*Lyrothorax*) *yoritomus* Bates, 1873 | GenBank | na | na | na | AB243450 | na |
| *P.* (*Melanius*) *corvinus* (Dejean, 1828) | GenBank | na | na | na | EU142453 | na |
| *P.* (*Melanius*) *noguchii* Bates, 1873 | GenBank | na | na | na | AB243486 | na |
| *P.* (*Metallophilus*) *interruptus* (Dejean, 1828) | GenBank | na | na | na | AB243487 | na |
| *P.* (*Micronialoe*) *bifoveolatus* Park, Known & Lafer, 1996 | GenBank | na | na | na | AB243458 | na |
| *P.* (*Morphnosoma*) *melanarius* (Illiger, 1798) | GenBank | na | na | na | AF398707 | AF002779 |
| *P.* (*Morphnosoma*) *stygicus* (Say, 1823) | GenBank | na | na | na | EU142448 | na |
| *P.* (*Morphnosoma*) *thunbergi* A. Morawitz, 1862 | GenBank | na | na | na | AB243455 | na |
| *P.* (*Nialoe*) *brunneipennis* Straneo, 1955 | GenBank | na | na | na | AB243501 | na |
| *P.* (*Oreophilus*) *flavofemoratus* (Dejean, 1828) | GenBank | na | na | na | AB243493 | na |
| *P.* (*Oreophilus*) *morio* (Duftschmid, 1812) | GenBank | na | na | na | AB243494 | na |
| *P.* (*Oreophilus*) *spinolae* (Dejean, 1828) | GenBank | na | na | na | AB243495 | na |
| *P.* (*Orsonjohnsonus*) *johnsoni* Ulke, 1889 | GenBank | na | na | na | EU142455 | na |
| *P.* (*Petrophilus*) *pertinax* (Tschitscherine, 1895) | GenBank | na | na | na | AB243448 | na |
| *P.* (*Phonias*) *diligens* (Sturm, 1824) | GenBank | na | na | na | AB243461 | na |
| *P.* (*Phonias*) *strenuus* (Panzer, 1796) | GenBank | na | na | na | EU142456 | na |
| *P.* (*Phonias*) *strenuus* (Panzer, 1796)^1^ | GERMANY: Rostock-Markgrafenheide, alt. 1 m | 54°13’  12°10’ | 15.XI.2009  J. Schmidt | hs1165 | hs1165 | hs1165 |
| *P.* (*Platypterus*) *truncatus* (Dejean, 1828) | GenBank | na | na | na | AB243492 | na |
| *P.* (*Platysma*) *leptis* Bates, 1873 | GenBank | na | na | na | AB243456 | na |
| *P.* (*Platysma*) *niger* Schaller, 1783^1^ | GERMANY, Mecklenburg, Ehbruch, alt. 3 m | 54°09’  11°58’ | 24.IX.2009  J. Schmidt | hs1143 | hs1143 | hs1143 |
| *P.* (*Pseudethira*) *angoarnigi pathibaraensis* Schmidt, 2012^8^ | NEPAL: Taplejung Distr., Pathibara Devi, alt. 3551 m | 27º26’  87º46’ | 16.VI.2011  S. Tamang | hs1168 | hs1168 | hs1168 |
| *P.* (*Pseudethira*) *balachowskyi* *anguleus* Morvan, 1972^6^ | NEPAL: Annapurna Himal, Dobate NW of Tadapani, alt. 3150 m | 28°24'  83°44' | 30.VI.2008  S. Tamang | hs839 | hs839 | na |
| *P.* (*Pseudethira*) *balachowskyi* *anguleus* Morvan, 1972^6^ | NEPAL: Dhaulagiri I Himal S-slope, Hile Kharka, alt. 3000-3100 m | 28°29'  83°34' | 9.V.2009  J. Schmidt | na | hs950 | na |
| *P.* (*Pseudethira*) *balachowskyi* *anguleus* Morvan, 1972^6^ | NEPAL: Dhaulagiri I Himal S-slope, Hile Kharka, alt. 3000-3100 m | 28°29'  83°34' | 9.V.2009  J. Schmidt | hs953 | na | na |
| *P.* (*Pseudethira*) *balachowskyi* *balachowskyi* Morvan, 1972^6^ | NEPAL: W-slope Annapurna Himal, Thulo Bugin Kharka, alt. 4200 m [locus typicus!] | 28°36'  83°41' | 1.VI.2004  J. Schmidt | hs512 | hs512 | hs512 |
| *P.* (*Pseudethira*) *balachowskyi* *balachowskyi* Morvan, 1972^6^ | NEPAL: SW-slope Dhaulagiri Himal, Kali Gandaki Valley, above Lete, alt. 2900 m | 28°37’  83°35' | 5.VI.2004  J. Schmidt | hs514 | na | na |
| *P.* (*Pseudethira*) *balachowskyi* *balachowskyi* Morvan, 1972^6^ | NEPAL: Muktinath Himal, above S of Muktinath, alt. 4000-4400 m | 28°47'  83°52' | 16.V.2007  J. Schmidt | hs564 | na | na |
| *P.* (*Pseudethira*) *balachowskyi* *balachowskyi* Morvan, 1972^6^ | NEPAL: Nilgiri Himal, Mesokanthu La Pass W-slope, alt. 4100-4300 m | 28°44'  83°48' | 18.V.2007  J. Schmidt | hs574 | na | na |
| *P.* (*Pseudethira*) *balachowskyi* *balachowskyi* Morvan, 1972^6^ | NEPAL: Nilgiri North Himal W-slope, above Sauru, alt. 3900-4200 m | 28°41'  83°40' | 9.VII.2008  S. Tamang | hs769 | na | na |
| *P.* (*Pseudethira*) *balachowskyi* *balachowskyi* Morvan, 1972^6^ | NEPAL: Nilgiri North Himal W-slope, above Sauru, alt. 3150 m | 28°40'  83°38' | 8.VII.2008  S. Tamang | hs792 | na | na |
| *P.* (*Pseudethira*) *balachowskyi dhaulagiricus* Straneo, 1983^6^ | NEPAL: foothills of Dhaulagiri Himal, 30 km W of Baglung, alt. 2800 m | 28°22'  83°20' | 19.V.2004  J. Schmidt | hs507 | na | na |
| *P.* (*Pseudethira*) *balachowskyi dhaulagiricus* Straneo, 1983^6^ | NEPAL: foothills of Dhaulagiri Himal, 30 km W of Baglung, alt. 2800 m | 28°22'  83°20' | 19.V.2004  J. Schmidt | na | hs508 | na |
| *P.* (*Pseudethira*) *balachowskyi* *myagdikholensis* Schmidt, 2006^6^ | NEPAL: Dhaulagiri I Himal S-slope, Lete Pass SW-slope, alt. 3900 m | 28°34’  83°33’ | 12.VII.2008  S. Tamang | hs746 | hs746 | na |
| *P.* (*Pseudethira*) *balachowskyi* *myagdikholensis* Schmidt, 2006^6^ | NEPAL: Dhaulagiri I Himal SW-slope, Lete pass, alt. 4050 m | 28°36’  83°34’ | 11.VII.2008  S. Tamang | hs790 | hs790 | na |
| *P.* (*Pseudethira*) *balachowskyi* *myagdikholensis* Schmidt, 2006^6^ | NEPAL: Dhaulagiri I Himal S-slope, Asnam Duri N-slope, alt. 3150-3400 m | 28°31'  83°27' | 14.V.2009  J. Schmidt | hs954 | hs954 | na |
| *P.* (*Pseudethira*) *balachowskyi* *myagdikholensis* Schmidt, 2006^6^ | NEPAL: Dhaulagiri I Himal S-slope, Asnam Duri SE-slope, alt. 3150 m | 28°31'  83°28' | 14.V.2009  J. Schmidt | hs1017 | na | na |
| *P.* (*Pseudethira*) *balachowskyi trapezicollis* Schmidt, 2006^6^ | NEPAL: Mardi Himal S-slope, above Korchon, alt. 3900 m | 28°25'  83°55' | 10.V.2008  J. Schmidt | hs642 | na | na |
| *P.* (*Pseudethira*) *balachowskyi trapezicollis* Schmidt, 2006^6^ | NEPAL: Mardi Himal S-slope, above Korchon, alt. 3900 m | 28°25'  83°55' | 10.V.2008  J. Schmidt | hs644 | na | na |
| *P.* (*Pseudethira*) *balachowskyi trapezicollis* Schmidt, 2006^6^ | NEPAL: Mardi Himal S-slope, Korchon Danda W-slope, alt. 2800 m | 28°23'  83°54' | 13.V.2008  J. Schmidt | hs676 | na | na |
| *P.* (*Pseudethira*) *balachowskyi trapezicollis* Schmidt, 2006^6^ | NEPAL: Mardi Himal S-slope, Korchon Danda W-slope, alt. 2800 m | 28°23'  83°54' | 13.V.2008  J. Schmidt | hs677 | na | na |
| *P.* (*Pseudethira*) *balachowskyi trapezicollis* Schmidt, 2006^6^ | NEPAL: Mardi Himal S-slope, Korchon Danda S-slope, alt. 3200 m | 28°23'  83°56' | 8.V.2008  J. Schmidt | hs689 | na | na |
| *P.* (*Pseudethira*) *balachowskyi trapezicollis* Schmidt, 2006^6^ | NEPAL: Mardi Himal S-slope, Korchon Danda W-slope, alt. 3100 m | 28°24'  83°54' | 12.V.2008  J. Schmidt | hs723 | na | na |
| *P.* (*Pseudethira*) *balachowskyi trapezicollis* Schmidt, 2006^6^ | NEPAL: Mardi Himal S-slope, Korchon Danda W-slope, alt. 3100 m | 28°24'  83°54' | 12.V.2008  J. Schmidt | hs724 | na | na |
| *P.* (*Pseudethira*) *balachowskyi trapezicollis* Schmidt, 2006^6^ | NEPAL: Mardi Himal S-slope, above Korchon, alt. 4300 m | 28°26'  83°56' | 19.VII.2008  S. Tamang | hs731 | na | na |
| *P.* (*Pseudethira*) *balachowskyi trapezicollis* Schmidt, 2006^6^ | NEPAL: SW-slope Mardi Himal, Low Camp, alt. 2800-3200 m | 28°23'  83°51' | 30.V.2011  S. Tamang | hs1177 | na | na |
| *P.* (*Pseudethira*) *balachowskyi trapezicollis* Schmidt, 2006^6^ | NEPAL: SW-slope Mardi Himal, Low Camp, alt. 2800-3200 m | 28°23'  83°51' | 30.V.2011  S. Tamang | hs1178 | na | na |
| *P.* (*Pseudethira*) *balachowskyi trapezicollis* Schmidt, 2006^6^ | NEPAL: SW-slope Mardi Himal, Low Camp, alt. 2800-3200 m | 28°23'  83°51' | 30.V.2011  S. Tamang | hs1179 | na | na |
| *P.* (*Pseudethira*) *balachowskyi tukchensis* Straneo, 1983^6^ | NEPAL: Dhaulagiri I Himal NE-slope, Kali Gandaki Valley, above Marpha, alt. 3800 m [locus typicus!] | 28°45'  83°39' | 20.V.2007  J. Schmidt | hs566 | hs566 | na |
| *P.* (*Pseudethira*) *balachowskyi tukchensis* Straneo, 1983^6^ | NEPAL: W-slope Dhaulagiri Himal, Kali Gandaki Valley, above Sokung, alt. 3800 m | 28°41'  83°34' | 7.VII.2008  S. Tamang | hs818 | na | na |
| *P.* (*Pseudethira*) *balu* Schmidt, 2009^7^ | NEPAL: Chakhure Lekh S-slope, above Gothgaon, alt. 2400-2500 m [locus typicus!] | 28°59'  82°17' | 17.VI.2009  S. Tamang | hs1020 | hs1020 | na |
| *P.* (*Pseudethira*) *balu* Schmidt, 2009^7^ | NEPAL: Chakhure Lekh S-slope, above Gothgaon, alt. 2400-2500 m [locus typicus!] | 28°59'  82°17' | 17.VI.2009  S. Tamang | na | hs1021 | na |
| *P.* (*Pseudethira*) *balu* Schmidt, 2009^7^ | NEPAL: Chakhure Lekh S-slope, Tantapauwa, alt. 3600 m | 29°05'  82°22' | 19.VI.2009  S. Tamang | hs1025 | hs1025 | na |
| *P.* (*Pseudethira*) cf. *thanglaensis* Schmidt, 2012^8^ | NEPAL: Taplejung Distr., Olangchung Gola, alt. 3720 m | 27º35’  87º41’ | 7.VII.2011  S. Tamang | hs1171 | hs1171 | na |
| *P.* (*Pseudethira*) *chainapaani* Schmidt, 2006^6^ | NEPAL: foothills of Dhaulagiri Himal, 30 km W of Baglung, alt. 2800 m [locus typicus!] | 28°22'  83°20' | 19.V.2004  J. Schmidt | hs520 | hs520 | hs520 |
| *P.* (*Pseudethira*) *chainapaani* Schmidt, 2006^6^ | NEPAL: Dhaulagiri I Himal S-slope, N of Banduk, alt. 2400-2600 m | 28°28'  83°35' | 8.V.2009  J. Schmidt | hs976 | hs976 | na |
| *P.* (*Pseudethira*) *chainapaani* Schmidt, 2006^6^ | NEPAL: Dhaulagiri I Himal S-slope, above Patlekharka, alt. 2500-2700 m | 28°32'  83°29' | 12.V.2009  J. Schmidt | hs992 | na | na |
| *P.* (*Pseudethira*) *chainapaani* Schmidt, 2006^6^ | NEPAL: Dhaulagiri I Himal S-slope, Pass Bagar Khola-Rahughat Khola, alt. 2600-2700 m | 28°29'  83°31' | 11.V.2009  J. Schmidt | hs1010 | na | na |
| *P.* (*Pseudethira*) *conaensis* Schmidt & Tian, 2011^9^; HOLOTYPE | CHINA: S Tibet, SSW Cona, 3km to Mama Xiang, alt. 3066 m | 27º54’  91º48’ | 25.VI.2010  M.-Y. Tian | hs1146 | hs1146 | hs1146 |
| *P.* (*Pseudethira*) *deuvei deuvei* Lassalle, 1985^7^ | NEPAL: Manaslu Himal SW-slope, upper Ngadi Khola Valley, alt. 2200 m | 28°22'  84°29' | 15.V.2005  J. Schmidt | hs579 | na | na |
| *P.* (*Pseudethira*) *deuvei deuvei* Lassalle, 1985^7^ | NEPAL: Manaslu Himal S-slope, S of Bara Pokhari, alt. 2150 m | 28°15'  84°25' | 1.V.2005  J. Schmidt | hs582 | hs582 | hs582 |
| *P.* (*Pseudethira*) *deuvei deuvei* Lassalle, 1985^7^ | NEPAL: Manaslu Himal SE-slope, W of Gupchi Danda, alt. 2250 m | 28°08'  84°44' | 18.V.2006  J. Schmidt | hs584 | hs584 | na |
| *P.* (*Pseudethira*) *deuvei hypocrita* Schmidt, 2009^7^ | NEPAL: Ganesh Himal S-slope, Pangsang Danda W slope, alt. 2550 m | 28°09'  85°07' | 3.V.2007  G. Hirthe | hs561 | hs561 | na |
| *P.* (*Pseudethira*) *deuvei hypocrita* Schmidt, 2009^7^ | NEPAL: Ganesh Himal S-slope, Pasamcho Danda, alt. 2300-2500 m [locus typicus!] | 28°01'  85°04' | 23.IV.2007  G. Hirthe | hs600 | hs600 | hs600 |
| *P.* (*Pseudethira*) *dhorpatanicus* Straneo, 1977^6^ | NEPAL: foothills of Dhaulagiri Himal, 30 km W of Baglung, alt. 2800 m | 28°22'  83°20' | 19.V.2004  J. Schmidt | hs518 | hs518 | hs518 |
| *P.* (*Pseudethira*) *dhorpatanicus* Straneo, 1977^6^ | NEPAL: foothills of Dhaulagiri Himal, 30 km W of Baglung, alt. 2800 m | 28°22'  83°20' | 19.V.2004  J. Schmidt | na | hs519 | na |
| *P.* (*Pseudethira*) *fritzhiekei* Schmidt, 1994^6^ | NEPAL: Lamjung Himal S-slope, East of Taunja Danda, alt. 3800-4200 m | 28°26'  84°15' | 3.VI.2007  S. Tamang | hs416 | hs416 | hs416 |
| *P.* (*Pseudethira*) *fritzhiekei* Schmidt, 1994^6^ | NEPAL: Lamjung Himal SE-slope, Sundar Danda S-slope, alt. 2800-3200 m | 28°22'  84°21' | 6.VI.2007  S. Tamang | hs620 | hs620 | na |
| *P.* (*Pseudethira*) *gagates delaroueriei* Morvan, 1994^6^ | NEPAL: Makalu Himal S-slope, N of Bhojpur, Jawabari, alt. 2450 m | na | 26.V.2008  S. Tamang | hs901 | na | na |
| *P.* (*Pseudethira*) *gagates delaroueriei* Morvan, 1994^6^ | NEPAL: Makalu Himal S-slope, N of Bhojpur, Jawabari, alt. 2450 m | na | 26.V.2008  S. Tamang | na | hs902 | na |
| *P.* (*Pseudethira*) *gagates delaroueriei* Morvan, 1994^6^ | NEPAL: Makalu Himal S-slope, N of Bhojpur, Belbati, alt. 2800 m | na | 22.V.2008  S. Tamang | hs922 | hs922 | hs922 |
| *P.* (*Pseudethira*) *gagates gagates* (Hope, 1831)^6^ | NEPAL: S-slope Shivapuri Lekh, W of Bagmati river, alt. 2100 m [locus typicus!] | 27°47’  85°24’ | 22.V.2005  J. Schmidt | hs581 | hs581 | hs581 |
| *P.* (*Pseudethira*) *ganesh atavus* Schmidt, 2006^10^ | NEPAL: Manaslu Himal NE-slope, above Prok, alt. 3000 m [locus typicus!] | 28°30’  84°49’ | 27.V.2006  J. Schmidt | hs590 | na | na |
| *P.* (*Pseudethira*) *ganesh atavus* Schmidt, 2006^10^ | NEPAL: Manaslu Himal N-slope, near Shyala, alt. 3300 m | 28°33’  84°41’ | 31.V.2006  J. Schmidt | na | hs592 | na |
| *P.* (*Pseudethira*) *ganesh atavus* Schmidt, 2006^10^ | NEPAL: Manaslu Himal N-slope, near Shyala, alt. 3300 m | 28°33’  84°41’ | 31.V.2006  J. Schmidt | hs593 | na | na |
| *P.* (*Pseudethira*) *ganesh ganesh* Schmidt, 2006^6^ | NEPAL: Ganesh Himal S-slope, Tiru Danda, alt. 3500-3700 m | 28°06'  85°08' | 29.IV.2007  G. Hirthe | hs596 | hs596 | hs596 |
| *P.* (*Pseudethira*) *ganesh ganesh* Schmidt, 2006^6^ | NEPAL: Ganesh Himal S-slope, Pansang Danda, alt. 3700-3900 m | 28°09'  85°10' | 30.IV.2007  G. Hirthe | hs602 | hs602 | na |
| *P.* (*Pseudethira*) *ganesh ganesh* Schmidt, 2006^6^ | NEPAL: Ganesh Himal S-slope, Pansang Danda, alt. 3700-3900 m | 28°09'  85°10' | 30.IV.2007  G. Hirthe | na | hs604 | na |
| *P.* (*Pseudethira*) *ganesh ganesh* Schmidt, 2006^6^ | NEPAL: Ganesh Himal S-slope, Sedre Danda, alt. 3200-3500 m | 28°04'  85°07' | 26.IV.2007  G. Hirthe | hs606 | hs606 | na |
| *P.* (*Pseudethira*) *ganja ganja* Schmidt, 1995^6^ | NEPAL: Lamjung Himal N-slope, above Temang, alt. 3000-3100 m [locus typicus!] | 28°30’  84°18’ | 6.V.2007  J. Schmidt | hs420 | hs420 | hs420 |
| *P.* (*Pseudethira*) *ganja ganja* Schmidt, 1995^6^ | NEPAL: Lamjung Himal N-slope, above Temang, alt. 3000-3100 m [locus typicus!] | 28°30’  84°18’ | 6.V.2007  J. Schmidt | hs421 | na | na |
| *P.* (*Pseudethira*) *ganja ganja* Schmidt, 1995^6^ | NEPAL: Lamjung Himal N-slope, above Temang, alt. 3000-3100 m [locus typicus!] | 28°30’  84°18’ | 6.V.2007  J. Schmidt | hs422 | na | na |
| *P.* (*Pseudethira*) *ganja ganja* Schmidt, 1995^6^ | NEPAL: Manaslu Himal N-slope, upper Dudh Khola Valley, alt. 3000-3100 m | 28°35'  84°26' | 4.VI.2006  J. Schmidt | hs490 | na | na |
| *P.* (*Pseudethira*) *ganja ganja* Schmidt, 1995^6^ | 3000-3100 m | 28°35'  84°26' | 4.VI.2006  J. Schmidt | hs491 | na | na |
| *P.* (*Pseudethira*) *ganja ganja* Schmidt, 1995^6^ | NEPAL: Manaslu Himal N-slope, upper Dudh Khola Valley, alt. 3000-3100 m | 28°35'  84°26' | 4.VI.2006  J. Schmidt | hs492 | na | na |
| *P.* (*Pseudethira*) *ganja ganja* Schmidt, 1995^6^ | NEPAL: Manaslu Himal SW-slope, upper Ngadi Khola Valley, alt. 3200 m | 28°21'  84°30' | 11.V.2005  J. Schmidt | hs495 | na | na |
| *P.* (*Pseudethira*) *ganja ganja* Schmidt, 1995^6^ | NEPAL: Manaslu Himal SW-slope, upper Ngadi Khola Valley, alt. 3200 m | 28°21'  84°30' | 11.V.2005  J. Schmidt | hs496 | na | na |
| *P.* (*Pseudethira*) *ganja ganja* Schmidt, 1995^6^ | NEPAL: Manaslu Himal S-slope, above Bara Pokhari, 3600-3900 m | 28°19'  84°30' | 7.V.2005  J. Schmidt | hs497 | na | na |
| *P.* (*Pseudethira*) *ganja ganja* Schmidt, 1995^6^ | NEPAL: Manaslu Himal S-slope, above Bara Pokhari, 3600-3900 m | 28°19'  84°30' | 7.V.2005  J. Schmidt | hs498 | hs498 | na |
| *P.* (*Pseudethira*) *ganja ganja* Schmidt, 1995^6^ | NEPAL: Manaslu Himal S-slope, above Bara Pokhari, 3600-3900 m | 28°19'  84°30' | 7.V.2005  J. Schmidt | hs499 | na | na |
| *P.* (*Pseudethira*) *ganja ganja* Schmidt, 1995^6^ | NEPAL: Manaslu Himal S-slope, above Bara Pokhari, 3600-3900 m | 28°19'  84°30' | 7.V.2005  J. Schmidt | hs500 | na | na |
| *P.* (*Pseudethira*) *ganja pisangensis*, Schmidt 2007^11^ | NEPAL: Annapurna II Himal N-slope, SE of Pisang, alt. 3200 m [locus typicus!] | 28°36’  84°10’ | 8.V.2007  J. Schmidt | hs423 | na | na |
| *P.* (*Pseudethira*) *ganja pisangensis*, Schmidt 2007^11^ | NEPAL: Annapurna II Himal N-slope, SE of Pisang, alt. 3200 m [locus typicus!] | 28°36’  84°10’ | 8.V.2007  J. Schmidt | hs424 | na | na |
| *P.* (*Pseudethira*) *ganja pisangensis*, Schmidt 2007^11^ | NEPAL: Annapurna II Himal N-slope, SE of Pisang, alt. 3200 m [locus typicus!] | 28°36’  84°10’ | 8.V.2007  J. Schmidt | hs425 | na | na |
| *P.* (*Pseudethira*) *gerdi gerdi* Schmidt, 2009^9^ | NEPAL: Saipal Himal S-slope, Ghatganga Khola 30 km NE Chainpur, alt. 2400 m [locus typicus!] | 28°01’  85°04’ | 25.VI.2009  A. Weigel | hs1070 | na | na |
| *P.* (*Pseudethira*) *gerdi gerdi* Schmidt, 2009^9^ | NEPAL: Saipal Himal S-slope, Ghatganga Khola 30 km NE Chainpur, alt. 2400 m [locus typicus!] | 28°01’  85°04’ | 25.VI.2009  A. Weigel | hs1071 | na | na |
| *P.* (*Pseudethira*) *gerdi khaptadensis* Schmidt, 2009^9^ | NEPAL: Doti Khaptad National Park, Khaptad Lake, alt. 3020 m [locus typicus!] | 29°23’  81°10’ | 2.VII.2009  A. Weigel | hs1072 | na | na |
| *P.* (*Pseudethira*) *gerdi khaptadensis* Schmidt, 2009^9^ | NEPAL: Doti Khaptad National Park, Khaptad Lake, alt. 3020 m [locus typicus!] | 29°23’  81°10’ | 2.VII.2009  A. Weigel | hs1073 | hs1073 | hs1073 |
| *P.* (*Pseudethira*) *gompanus* Straneo, 1983^6^ | NEPAL: Dolpo, Bhalu Himal E-slope, Bhalu Khola W of Sharmi, alt. 2800 m | 29°04’  82°28’ | 1.X.2007  S. Tamang | hs552 | hs552 | hs552 |
| *P.* (*Pseudethira*) *hartmanni hartmanni* Schmidt, 1995^6^ | NEPAL: Lamjung Himal N-slope, Temang, alt. 2550 m [locus typicus!] | 28°31’  84°18’ | 5.V.2007  J. Schmidt | hs560 | hs560 | hs560 |
| *P.* (*Pseudethira*) *hartmanni khukuri* Schmidt, 2006^6^ | NEPAL: Lamjung Himal SW-slope, Bachhar Kharka NE of Sikles, alt. 2100-2300 m | 28°22'  84°08' | 31.V.2007  S. Tamang | hs621 | hs621 | na |
| *P.* (*Pseudethira*) *hoelli* Schmidt, 2009^9^ | NEPAL: S-slope Chakhure Lekh, above Gothgaon, alt. 2400-2500 m | 28°59'  82°17' | 17.VI.2009  S. Tamang | hs1022 | hs1022 | na |
| *P.* (*Pseudethira*) *immarginatus* Straneo, 1977^6^ | NEPAL: SW Gosainkund Lekh, NE above Gumbadanda, alt. 2600-2900 m | 28°02'  85°14' | 25.V.2009  S. Tamang | hs1034 | hs1034 | hs1034 |
| *P.* (*Pseudethira*) *jaljaleensis* Schmidt, 2009^8^ | NEPAL: Jaljale Himal, 3600 m | na | 30.VI.2009  P. Rai | hs1043 | hs1043 | na |
| *P.* (*Pseudethira*) *jaljaleensis* Schmidt, 2009^8^ | NEPAL: Taplejung Distr., Jaljale Pokhari, alt. 4200 m | 27º27’  87º27’ | 4.VI.2010  S. Tamang | hs1175 | na | na |
| *P.* (*Pseudethira*) *jaljaleensis* Schmidt, 2009^8^ | NEPAL: Taplejung Distr., Jaljale Pokhari, alt. 4200 m | 27º27’  87º27’ | 4.VI.2010  S. Tamang | hs1176 | hs1176 | na |
| *P.* (*Pseudethira*) *janbritoi tamur* Schmidt, 2012^8^ | NEPAL: Taplejung Distr., Maiwa Danda, alt. 2660 m | 27º28’  87º38’ | 20.VI.2010  S. Tamang | hs1167 | hs1167 | na |
| *P.* (*Pseudethira*) *juga* Schmidt, 2006^6^ | NEPAL: W-slope Annapurna Himal, below Thulo Bugin, above Dana, alt. 2850 m | 28°33’  83°39’ | 29.V.2004  J. Schmidt | hs535 | hs535 | hs535 |
| *P.* (*Pseudethira*) *juga* Schmidt, 2006^6^ | NEPAL: S-slope Annapurna II Himal, above Garlang, alt. 2000-2200 m | 28°18’  83°59’ | 29.V.2007  S. Tamang | hs630 | hs630 | na |
| *P.* (*Pseudethira*) *juga* Schmidt, 2006^6^ | NEPAL: S-slope Annapurna II Himal, above Garlang, alt. 2000-2200 m | 28°18’  83°59’ | 29.V.2007  S. Tamang | na | hs631 | na |
| *P.* (*Pseudethira*) *juga* Schmidt, 2006^6^ | NEPAL: S-slope Dhaulagiri I Himal, N of Banduk, alt. 1900-2300 m | 28°28'  83°35' | 6-7.V.2009  J. Schmidt | hs996 | hs996 | na |
| *P.* (*Pseudethira*) *juga* Schmidt, 2006^6^ | NEPAL: Mardi Himal, Deurali, alt. 21-2300 m | 28°19' 83°50' | 29.V.2011  S. Tamang | hs1183 | hs1183 | na |
| *P.* (*Pseudethira*) *kalo* Schmidt, 2012^8^ | NEPAL: Taplejung Distr., Maiwa Danda, alt. 2660 m | 27º28’  87º38’ | 20.VI.2011  S. Tamang | hs1166 | hs1166 | hs1166 |
| *P.* (*Pseudethira*) *kleinfeldi* Straneo, 1982^6^ | NEPAL: S-slope Annapurna South Himal, above Shika village, alt. 2100 m | 28°26’  83°41’ | 27.V.2004  J. Schmidt | hs542 | hs542 | hs542 |
| *P.* (*Pseudethira*) *kleinfeldi* Straneo, 1982^6^ | NEPAL: S-slope Annapurna II Himal, above Sikles village, alt. 2000-2400 m | 28°22’  84°07’ | S. Tamang  30.V.2007 | hs555 | hs555 | na |
| *P.* (*Pseudethira*) *kleinfeldi* Straneo, 1982^6^ | NEPAL: SW-slope Lamjung Himal, Bachhar Kharka NE Sikles, alt. 2100-2300 m | 28°22’  84°08’ | S. Tamang  31.V.2007 | hs624 | hs624 | na |
| *P.* (*Pseudethira*) *kleinfeldi* Straneo, 1982^6^ | NEPAL: SW-slope Lamjung Himal, Bachhar Kharka NE Sikles, alt. 2100-2300 m | 28°22’  84°08’ | S. Tamang  31.V.2007 | hs626 | na | na |
| *P.* (*Pseudethira*) *kleinfeldi* Straneo, 1982^6^ | NEPAL: SE-slope Lamjung Himal, S-slope Sundar Danda, alt. 2200-2500 m | 28°20’  84°22’ | S. Tamang  7.VI.2007 | hs632 | hs632 | na |
| *P.* (*Pseudethira*) *kleinfeldi* Straneo, 1982^6^ | NEPAL: Mardi Himal, W-slope Korchon Danda, alt. 2300 m | 28°23’  84°54’ | 14.V.2008  J. Schmidt | hs662 | hs662 | na |
| *P.* (*Pseudethira*) *kleinfeldi* Straneo, 1982^6^ | NEPAL: Mardi Himal, Deurali, alt. 21-2300 m | 28°19' 83°50' | 29.V.2011  S. Tamang | hs1180 | na | na |
| *P.* (*Pseudethira*) *kleinfeldi* Straneo, 1982^6^ | NEPAL: Mardi Himal, Deurali, alt. 21-2300 m | 28°19' 83°50' | 29.V.2011  S. Tamang | hs1181 | hs1181 | hs1181 |
| *P.* (*Pseudethira*) *kleinfeldi* Straneo, 1982^6^ | NEPAL: Mardi Himal, Deurali, alt. 21-2300 m | 28°19' 83°50' | 29.V.2011  S. Tamang | hs1182 | hs1182 | na |
| *P.* (*Pseudethira*) *kleinfeldi* Straneo, 1982^6^ | NEPAL: Mardi Himal, Deurali, alt. 21-2300 m | 28°19' 83°50' | 29.V.2011  S. Tamang | hs1184 | hs1184 | na |
| *P.* (*Pseudethira*) *letensis* Habu, 1973^6^ | NEPAL: S-slope Annapurna South Himal, Ghorepani, alt. 2700-2800 m | 28°24’  83°42’ | 26.V.2004  J. Schmidt | hs506 | hs506 | hs506 |
| *P.* (*Pseudethira*) *letensis* Habu, 1973^6^ | NEPAL: W-slope Annapurna Himal, below Thulo Bugin, above Dana, alt. 2850 m | 28°33’  83°39’ | 29.V.2004  J. Schmidt | hs534 | na | na |
| *P.* (*Pseudethira*) *letensis* Habu, 1973^6^ | NEPAL: W-slope Dhaulagiri Himal, above place Lete, alt. 2700-2900 m [locus typicus!] | 28°37’  83°35' | 4.VI.2004  J. Schmidt | na | hs536 | na |
| *P.* (*Pseudethira*) *letensis* Habu, 1973^6^ | NEPAL: W-slope Dhaulagiri Himal, above place Lete, alt. 2700-2900 m [locus typicus!] | 28°37’  83°35' | 4.VI.2004  J. Schmidt | hs538 | na | na |
| *P.* (*Pseudethira*) *letensis* Habu, 1973^6^ | NEPAL: W-slope Dhaulagiri Himal, Kali Gandaki Valley, place Sokung, alt. 2700 m | 28°40'  83°35' | 6.VII.2008  S. Tamang | hs740 | na | na |
| *P.* (*Pseudethira*) *letensis* Habu, 1973^6^ | NEPAL: Nilgiri North Himal W-slope, above Sauru, alt. 3150 m | 28°40'  83°38' | 8.VII.2008  S. Tamang | hs795 | na | na |
| *P.* (*Pseudethira*) *letensis* Habu, 1973^6^ | NEPAL: W-slope Annapurna South Himal, NW-slope Kopra Danda, place Site Puing, alt. 2800 m | 28°28'  83°41' | 3.VII.2008  S. Tamang | hs856 | na | na |
| *P.* (*Pseudethira*) *letensis* Habu, 1973^6^ | NEPAL: W-slope Annapurna South Himal, NW-slope Kopra Danda, place Site Puing, alt. 2800 m | 28°28'  83°41' | 3.VII.2008  S. Tamang | hs857 | na | na |
| *P.* (*Pseudethira*) *letensis* Habu, 1973^6^ | NEPAL: S-slope Dhaulagiri Himal, upper Rahughat Khola valley, alt. 2750-3100 m | 28°34'  83°32' | 12.VII.2008  S. Tamang | hs822 | na | na |
| *P.* (*Pseudethira*) *letensis* Habu, 1973^6^ | NEPAL: Dhaulagiri I Himal S-slope, upper Marang Khola Valley, alt. 2500-2700 m | 28°29'  83°27' | 16.V.2009  J. Schmidt | hs970 | hs970 | na |
| *P.* (*Pseudethira*) *letensis* Habu, 1973^6^ | NEPAL: Dhaulagiri I Himal S-slope, N of Banduk, alt. 2400-2600 m | 28°28'  83°35' | 8.V.2009  J. Schmidt | hs974 | na | na |
| *P.* (*Pseudethira*) *letensis* Habu, 1973^6^ | NEPAL: Dhaulagiri I Himal S-slope, above Patlekharka, alt. 2500-2700 m | 28°32'  83°29' | 12.V.2009  J. Schmidt | hs988 | hs988 | na |
| *P.* (*Pseudethira*) *letensis* Habu, 1973^6^ | NEPAL: Dhaulagiri I Himal S-slope, N of Banduk, alt. 1900-2300 m | 28°28'  83°35' | 6-7.V.2009  J. Schmidt | hs998 | hs998 | na |
| *P.* (*Pseudethira*) *letensis* Habu, 1973^6^ | NEPAL: Dhaulagiri I Himal S-slope, Pass Bagar Khola-Rahughat Khola, alt. 2600-2700 m | 28°29'  83°31' | 11.V.2009  J. Schmidt | hs1007 | na | na |
| *P.* (*Pseudethira*) *letensis* Habu, 1973^6^ | NEPAL: Dhaulagiri I Himal S-slope, Pass Bagar Khola-Rahughat Khola, alt. 2600-2700 m | 28°29'  83°31' | 11.V.2009  J. Schmidt | hs1009 | na | na |
| *P.* (*Pseudethira*) *letensis* Habu, 1973^6^ | NEPAL: Dhaulagiri I Himal S-slope, Bagar Khola, alt. 2250 m | 28°30'  83°32' | 10.V.2009  J. Schmidt | hs1011 | na | na |
| *P.* (*Pseudethira*) *matsumurai* Habu, 1973^6^ | NEPAL: Thare Danda, Gyaje Kharka, alt. 3900-4000 m | 28°04'  85°27' | 4.VI.2009  S. Tamang | hs1039 | hs1039 | hs1039 |
| *P.* (*Pseudethira*) *mewakholensis* Schmidt, 2012^8^ | NEPAL: Taplejung Distr., Mewa Khola, alt. 2970 m | 27º35’  87º35’ | 23.VI.2011  S. Tamang | hs1169 | hs1169 | na |
| *P.* (*Pseudethira*) *mewakholensis* Schmidt, 2012^8^ | NEPAL: Taplejung Distr., Tamur river, alt. 3130 m | 27º34’  87º40’ | 8.VII.2011  S. Tamang | hs1170 | hs1170 | na |
| *P.* (*Pseudethira*) *nepalensis* Straneo, 1977^6^ | NEPAL: S-slope Kanjiroba Himal, E of Jumla, Gothichaur pass, alt. 3000 m | 29°13’  82°18’ | 28.IX.2007  S. Tamang | hs418 | hs418 | na |
| *P.* (*Pseudethira*) *nepalensis* Straneo, 1977^6^ | NEPAL: Dolpo, Bhalu Himal N-slope, NW of Bhalu Pass, alt. 3500 m | 29°09'  82°23' | 30.IX.2007  S. Tamang | hs544 | hs544 | na |
| *P.* (*Pseudethira*) *nepalensis* Straneo, 1977^6^ | NEPAL: S-slope Kanjiroba Himal, pass Muria Lagna E-slope, alt. 3450 m | 29°08'  82°31' | 14.X.2007  S. Tamang | hs551 | na | na |
| *P.* (*Pseudethira*) *nepalensis* Straneo, 1977^6^ | NEPAL: Chakhure Lekh, Chakhure Bhanjyang N-slope, alt. 3200-3300 m | 29°08'  82°22' | 21.VI.2009  S. Tamang | hs1028 | na | na |
| *P.* (*Pseudethira*) *nepalensis* Straneo, 1977^6^ | NEPAL: Jumla Distr., Bhadali to Bhadali Pass, alt. 3140-3610 m | 29°20'  82°09' | 13.VI.2011  M. Hartmann | hs1186 | hs1186 | hs1186 |
| *P.* (*Pseudethira*) *nepalensis* Straneo, 1977^6^ | NEPAL: Jumla Distr., Bhadali to Bhadali Pass, alt. 3140-3610 m | 29°20'  82°09' | 13.VI.2011  M. Hartmann | hs1187 | na | na |
| *P.* (*Pseudethira*) *olafi* Schmidt, 2006^6^ | NEPAL: S-slope Annapurna South Himal, Banthanti, alt. 2300-2400 m [locus typicus!] | 28°22'  83°43' | 26.V.2004  J. Schmidt | hs540 | hs540 | hs540 |
| *P.* (*Pseudethira*) *pseudoharmandi* Morvan, 1981^8^ | NEPAL: S of Jaljale Himal, Basantapur, alt. 2100 m | 27°07'  87°25' | 13.VI.2009  P. Rai | hs1047 | hs1047 | hs1047 |
| *P.* (*Pseudethira*) *pseudoharmandi* Morvan, 1981^8^ | NEPAL: S of Jaljale Himal, Milke Danda, alt. 3000-3100 m | 27°23'  87°27' | 16.VI.2009  P. Rai | hs1054 | na | na |
| *P.* (*Pseudethira*) *schrettenbrunneri* Schmidt, 2012^8^ | NEPAL: Makalu Himal S-slope, Salpa Pass, alt. 3400 m | na | 31.V.2008  S. Tamang | hs878 | hs878 | hs878 |
| *P.* (*Pseudethira*) *schrettenbrunneri* Schmidt, 2012^8^ | NEPAL: Makalu Himal S-slope, N of Bhojpur, Belbati, alt. 2800 m | na | 22.V.2008  S. Tamang | hs923 | hs923 | na |
| *P.* (*Pseudethira*) *tulobalu* Schmidt, 2009^9^ | NEPAL: Chakhure Lekh S-slope, above Gothgaon, alt. 2400-2500 m [locus typicus!] | 28°59'  82°17' | 17.VI.2009  S. Tamang | hs1018 | hs1018 | na |
| *P.* (*Pseudethira*) *tulobalu* Schmidt, 2009^9^ | NEPAL: Chakhure Lekh S-slope, above Gothgaon, alt. 2400-2500 m [locus typicus!] | 28°59'  82°17' | 17.VI.2009  S. Tamang | na | hs1019 | na |
| *P.* (*Pseudoferonia*) *campbelli* Bousquet, 1985 | GenBank | na | na | na | EU142457 | na |
| *P. (Pseudohaptoderus) semenowi* (Tschitschérine, 1888)^12^ | CHINA: Central Tibet, Nyainqentanglha Shan N-slope to Namtso, alt. 4900 m | 30°38’  90°51’ | 13.VII.2007  J. Schmidt | hs459 | na | na |
| *P. (Pseudohaptoderus) semenowi* (Tschitschérine, 1888)^12^ | CHINA: Central Tibet, Nyainqentanglha Shan N-slope to Namtso, alt. 4900 m | 30°38’  90°51’ | 13.VII.2007  J. Schmidt | na | hs460 | hs460 |
| *P. (Pseudohaptoderus) semenowi* (Tschitschérine, 1888)^12^ | CHINA : Qinghai Prov., Nangqeng, San Ziang Yuan N.R., alt. 3900-4200m | 31°52'  96°33' | 16.VI.2010  J. Martens | hs1137 | hs1137 | na |
| *P. (Pseudomaseus) ambigenus* Bates*,* 1883 | GenBank | na | na | na | AB243472 | na |
| *P. (Pseudomaseus) fuscicornis* (Reiche & Saulcy*,* 1855)^15^ | IRAN: Mazandaran Prov., vic. Now Shar, alt. 880 m | 36°31'  51°38' | 2.V.2010  A. Weigel | na | hs1140 | na |
| *P. (Pseudomaseus) luctuosus* (Dejean, 1828) | GenBank | na | na | na | EU142458 | na |
| *P. (Pseudomaseus) minor* (Gyllenhal, 1827)^1^ | GERMANY: Mecklenburg, Göldenitzer Moor, alt. 36 m | 54°00’  12°20’ | 15.VII.2009  J. Schmidt | hs1142 | hs1142 | hs1142 |
| *P. (Pseudomaseus) rhaeticus* Heer, 1837^1^ | GERMANY: Mecklenburg, Ivendorfer Forst, alt. 80 m | 54°03’  11°54’ | 15.V.2009  J. Schmidt | hs1141 | hs1141 | hs1141 |
| *P. (Pseudomaseus) rotundangulus* A. Morawitz, 1862 | GenBank | na | na | na | AB243451 | na |
| *P.* (*Pterostichus*) *fasciatopunctatus* (Creutzer, 1799)^1^ | GERMANY: Bavaria, Leimbach, alt. 890 m | 10°96'  47°65' | 27.VIII.2008  W. Lorenz | hs907 | hs907 | hs907 |
| *P.* (*Pterostichus*) *rutilans* (Dejean, 1828) | GenBank | na | na | na | AB243491 | na |
| *P.* (*Rhagadus*) *microcephalus* (Motschulsky, 1860) | GenBank | na | na | na | AB243454 | na |
| *P.* (*Sinosteropus*) *perhoplites* Schmidt & Tian, 2011^9^ | CHINA: S Tibet, SSW Cona, alt. 4465 m | 27º55’  91º52’ | 24.VI.2010  M.-Y. Tian | hs1148 | hs1148 | hs1148 |
| *P.* (*Sinosteropus*) *pseudojugivagus* Schmidt & Tian, 2011^9^ | CHINA: S-Tibet, Xuebudala Shan, pass, alt. 5108 m | 28º38’  92º13’ | 22.VI.2010  M.-Y. Tian | na | hs1150 | na |
| *P.* (*Sinosteropus*) *pseudosinensis pseudosinensis* Sciaky & Facchini, 2003^13^ | CHINA: Qinghai Prov., S Nangqeng, alt. 3950 m | 32°03'  96°31' | 16.VI.2010  J. Martens | hs1136 | hs1136 | hs1136 |
| *P.* (*Steropus*) *ebenus* (Quensel in Schönherr, 1806) = *globosus* (Fabricius, 1792) | GenBank | na | na | FJ173234 | na | FJ173132 |
| *P.* (unplaced) *deuvesianus* Morvan, 1995^15^ | NEPAL: S-slope Jaljale Himal, Milke Danda, Dobate, alt. 3500 m | na | 18.VI.2009  P. Rai | hs1057 | hs1057 | hs1057 |
| *P.* (unplaced) cf. *migliaccioi* Straneo, 1982 ^15^ | NEPAL: S-slope Makalu Himal, N of Bhojpur, Lawari Kharka, alt. 3250 m | na | 3.VI.2008  S. Tamang | na | hs910 | na |
| *Stomis pumicatus* (Panzer, 1796) | GenBank | na | na | na | EU142459 | na |
| *Synuchus vivalis* (Illiger, 1798) | GenBank | na | na | na | na | FJ173120 |
| *Tapinopterus balcanicus* Ganglbauer, 1891 | GenBank | na | na | na | EU142460 | na |
| *Trigonognatha* spec. | CHINA: E Tibet, Rawu Lake, alt. 3940 m | 29°29’  96°46’ | 10.VII.2009  M.-Y. Tian | hs1132 | hs1132 | hs1132 |
| *Trigonotoma morvani morvani* Deuve & Lassalle, 1983^14^ | NEPAL: S-slope Shivapuri Lekh, W of Bagmati river, alt. 2100 m | 27°47’  85°24’ | 22.V.2005  J. Schmidt | hs637 | hs637 | hs637 |
