# Supplementary material for: Into the Himalayan Exile: The Phylogeography of the Ground Beetle Ethira clade Supports the Tibetan Origin of Forest-Dwelling Himalayan Species Groups
Source: PLoS One. 2012 Sep 26;7(9):e45482. doi: 10.1371/journal.pone.0045482 (PMC3458877; doi:10.1371/journal.pone.0045482)
Supplement: Table S2 — Primers used for DNA amplification (amp) and sequencing (seq). (DOCX) [file pone.0045482.s007.docx]

**Table S2** Primers used for DNA amplification (amp) and sequencing (seq).

| **Primer** |  | **Direction** | **Sequence** | **Reference** |
| --- | --- | --- | --- | --- |
| **COI** |  |  |  |  |
| LCO1490 | amp+seq | forward | 5’-GGT CAA CAA ATC ATA AAG ATA TTG G-3’ | [72] |
| HCO709 | amp | reverse | 5’-AAT NAG AAT NTA NAC TTC NGG GTG-3’ | [73] |
| PterRevNew | amp+seq | reverse | 5’-CCT GTA TTR GCW GGR GCW ATT AC-3’ | this study |
| Co1RevEthira | amp | reverse | 5’-CCA ATA GCT AAT ATA GCA TAR ATT ATW CCT-3’ | this study |
| PterFw | amp+seq | forward | 5’-AGG AGC TCC TGA TAT AGC TTT-3’ | this study |
| KSCOInew | amp+seq | reverse | 5’-GGA GCA GTA TTT GCT ATT ATA GCA-3’ | this study |
| JER | amp+seq | forward | 5’-CAA CAT TTA TTT TGA TTT TTT GG-3’ | [74] |
| PATnew | amp+seq | reverse | 5’- TCT AAT ATG GCA GAW TAG TGC AHT-3’ | this study |
| **28S rDNA** |  |  |  |  |
| D1 | amp+seq | forward | 5’-GGG AGG AAA AGA AAC TAA C-3’ | [75] |
| D3i | amp+seq | reverse | 5’-GCA TAG TTC ACC ATC TTT C-3’ | [30] |
| int28SfwEthira | amp+seq | forward | 5’-GTATATAGCCGGAGGCGTGCAC-3’ | this study |
| int28SrevEthira | amp+seq | reverse | 5’-CATTTGTCACAAGTCTAAACGCTC-3’ | this study |
| **18S rDNA** |  |  |  |  |
| 18Sfw | amp+seq | forward | 5’-CCTAYCTGGTTGATCCTGCCAGT-3’ | [76] |
| 18Srev | amp+seq | reverse | 5’-TAATGATCCTTCCGCAGGTT-3’ | [76] |
| 18F509 | amp+seq | forward | 5’-CCCCGTAATTGGAATGAGTACA-3’ | [77] |
| 18F997 | amp+seq | forward | 5’-TTCGAAGACGATCAGATACCG-3’ | [77] |
| 18R1256 | amp | reverse | 5’-AGCTCTCAATCTGTCAATCCT-3’ | [77] |
| 18L | amp+seq | reverse | 5’-GAATTACCGCGGCTGCTGGCACC-3’ | [78] |
|  |  |  |  |  |
