# Supplementary material for: Into the Himalayan Exile: The Phylogeography of the Ground Beetle Ethira clade Supports the Tibetan Origin of Forest-Dwelling Himalayan Species Groups
Source: PLoS One. 2012 Sep 26;7(9):e45482. doi: 10.1371/journal.pone.0045482 (PMC3458877; doi:10.1371/journal.pone.0045482)
Supplement: Table S3 — PCR thermal cycling condition used. (DOCX) [file pone.0045482.s008.docx]

**Table S3** PCR thermal cycling condition used.

| **primer combination** | **Mg^2+^ finale concentration (mM)** | **number of cycles** | **annealing temperature (°C)** | **time of extension phase (s)** |
| --- | --- | --- | --- | --- |
| **COI** |  |  |  |  |
| LCO1490 / HCO709 | 3.5 | 38 | 50 | 60 |
| LCO1490 / PterRevNew | 3.5 | 38 | 50 | 60 |
| LCO1490 / PATnew | 3.5 | 38 | 52.5 | 200 |
| PterFw / KSCOInew | 3.5 | 38 | 50 | 60 |
| PterFw / COIrevEthira | 3.5 | 38 | 50 | 60 |
| JER / PATnew | 1.5 | 38 | 53 | 55 |
| **28S rDNA** |  |  |  |  |
| D1 / D3i | 2.5 | 38 | 51 | 55 |
| D1 / int28SrevEthira | 2.5 | 38 | 51 | 55 |
| int28SfwEthira / D3i | 2.5 | 38 | 51 | 55 |
| **18S rDNA** |  |  |  |  |
| 18Sfw / 18Srev | 1.5 | 38 | 52.5 | 200 |
| 18Sfw / 18R1256 | 1.5 | 38 | 52.5 | 200 |
| 18Sfw / 18L | 1.5 | 38 | 52.5 | 200 |
| 18F509 / 18R1296 | 1.5 | 38 | 52.5 | 200 |
| 18F997 / 18Srev | 1.5 | 38 | 52.5 | 200 |
