# Supplementary material for: Into the Himalayan Exile: The Phylogeography of the Ground Beetle Ethira clade Supports the Tibetan Origin of Forest-Dwelling Himalayan Species Groups
Source: PLoS One. 2012 Sep 26;7(9):e45482. doi: 10.1371/journal.pone.0045482 (PMC3458877; doi:10.1371/journal.pone.0045482)
Supplement: Table S4 — Divergence time estimates for the Ethira clade, the Euro-Alpine Pterostichus clade, and for the genus Pterostichus from five different calibration schemes (details see main text). (DOCX) [file pone.0045482.s009.docx]

**Table S4** Divergence time estimates for the *Ethira* clade, the Euro-Alpine *Pterostichus* clade, and for the genus *Pterostichus* from five different calibration schemes (details see main text).

| **Rates, Pterostichus** | **tmrca** | | | **ucld** | |  |
| --- | --- | --- | --- | --- | --- | --- |
| **& Euro-Alpine** | **Ethira** | **Euro-Alpine** | **Pterostichus** | **mean** | **stdev** | **meanRate** |
| **mean** | 19.1294 | 12.4345 | 36.5645 | 3.10E-03 | 0.6026 | 3.81E-03 |
| **stderr of mean** | 0.1921 | 4.01E-02 | 6.88E-03 | 2.46E-05 | 6.73E-03 | 2.03E-05 |
| **median** | 18.658 | 12.3813 | 35.9919 | 3.05E-03 | 0.5987 | 3.78E-03 |
| **95% HPD lower** | 11.9374 | 7.893 | 35.0252 | 2.13E-03 | 0.3434 | 2.81E-03 |
| **95% HPD upper** | 27.0643 | 17.0916 | 39.8678 | 4.19E-03 | 0.8714 | 4.88E-03 |
|  |  |  |  |  |  |  |
| **Pterostichus** | **tmrca** | | | **ucld** | |  |
| **& Euro-Alpine** | **Ethira** | **Euro-Alpine** | **Pterostichus** | **mean** | **stdev** | **meanRate** |
| **mean** | 18.3712 | 12.7088 | 36.4839 | 2.66E-03 | 0.4716 | 3.10E-03 |
| **stderr of mean** | 0.1538 | 7.87E-02 | 5.09E-02 | 2.04E-05 | 4.67E-03 | 1.76E-05 |
| **median** | 18.0514 | 12.6581 | 35.98 | 2.63E-03 | 0.4697 | 3.07E-03 |
| **95% HPD lower** | 12.286 | 7.9797 | 35.0459 | 1.85E-03 | 0.2917 | 2.35E-03 |
| **95% HPD upper** | 25.4162 | 17.1494 | 39.3704 | 3.52E-03 | 0.6783 | 3.95E-03 |
|  |  |  |  |  |  |  |
| **Euro-Alpine** | **tmrca** | | | **ucld** | |  |
|  | **Ethira** | **Euro-Alpine** | **Pterostichus** | **mean** | **stdev** | **meanRate** |
| **mean** | 20.7861 | 12.6399 | 39.7839 | 3.04E-03 | 0.4868 | 3.59E-03 |
| **stderr of mean** | 0.4741 | 0.1025 | 0.5459 | 1.55E-04 | 6.07E-03 | 1.84E-04 |
| **median** | 19.3136 | 12.7672 | 37.8867 | 2.52E-03 | 0.4856 | 2.96E-03 |
| **95% HPD lower** | 4.7424 | 5.6244 | 13.0501 | 9.34E-04 | 0.2908 | 1.01E-03 |
| **95% HPD upper** | 40.6271 | 19.8813 | 73.5539 | 5.69E-03 | 0.6677 | 6.46E-03 |
|  |  |  |  |  |  |  |
| **Pterostichus** | **tmrca** | | | **ucld** | |  |
|  | **Ethira** | **Euro-Alpine** | **Pterostichus** | **mean** | **stdev** | **meanRate** |
| **mean** | 18.9964 | 9.8565 | 36.3936 | 3.17E-03 | 0.6183 | 3.89E-03 |
| **stderr of mean** | 0.174 | 8.62E-02 | 5.45E-03 | 2.47E-05 | 6.69E-03 | 1.89E-05 |
| **median** | 18.628 | 9.5914 | 35.9025 | 3.13E-03 | 0.6156 | 3.85E-03 |
| **95% HPD lower** | 11.8605 | 4.4666 | 35.0278 | 2.15E-03 | 0.3657 | 2.86E-03 |
| **95% HPD upper** | 26.7709 | 15.7188 | 39.2344 | 4.28E-03 | 0.8796 | 4.95E-03 |
|  |  |  |  |  |  |  |
| **Rates** | **tmrca** | | | **ucld** | |  |
|  | **Ethira** | **Euro-Alpine** | **Pterostichus** | **mean** | **stdev** | **meanRate** |
| **mean** | 5.695 | 2.822 | 10.694 | 0.015 | 0.646 | 0.019 |
| **stderr of mean** | 0.213 | 0.136 | 0.474 | 0.000 | 0.006 | 0.000 |
| **median** | 4.089 | 1.978 | 7.642 | 0.014 | 0.646 | 0.018 |
| **95% HPD lower** | 1.221 | 0.367 | 2.265 | 0.002 | 0.395 | 0.002 |
| **95% HPD upper** | 13.636 | 7.047 | 25.791 | 0.028 | 0.898 | 0.036 |
